# Electrochromic-Induced Rechargeable Aqueous Batteries: An Integrated Multifunctional System for Cross-Domain Applications

**DOI:** 10.1007/s40820-023-01056-y

**Published:** 2023-04-07

**Authors:** Qi Zhao, Zhenghui Pan, Binbin Liu, Changyuan Bao, Ximeng Liu, Jianguo Sun, Shaorong Xie, Qing Wang, John Wang, Yanfeng Gao

**Affiliations:** 1 Department of Materials Science and Engineering, Shanghai University, Shanghai, 200444 People’s Republic of China; 2 Department of Materials Science and Engineering, National University of Singapore, Singapore, 117574 Singapore; 3 Department of Materials Science and Engineering, Tongji University, Shanghai, 200092 People’s Republic of China; 4 Department of Computer Engineering and Science, Shanghai University, Shanghai, 200444 People’s Republic of China; 5 National University of Singapore (Chongqing) Research Institute, Chongqing, 401120 People’s Republic of China; 6 Institute of Materials Research and Engineering, A*Star, Singapore, 138634 Singapore; 7 Key Laboratory of Comprehensive and Highly Efficient Utilization of Salt Lake Resources, Qinghai Institute of Salt Lakes, Chinese Academy of Sciences, Xining, 810008 People’s Republic of China

**Keywords:** Electrochromic, Aqueous batteries, Multifunctional, Integration

## Abstract

Multifunctional electrochromic-induced rechargeable aqueous batteries (MERABs) integrate electrochromism and aqueous ion batteries into one platform, which is able to deliver the conversion and storage of photo-thermal-electrochemical sources. Aqueous ion batteries compensate for the drawbacks of slow kinetic reactions and unsatisfied storage capacities of electrochromic devices. On the other hand, electrochromic technology can enable dynamically regulation of solar light and heat radiation. However, MERABs still face several technical issues, including a trade-off between electrochromic and electrochemical performance, low conversion efficiency and poor service life. In this connection, novel device configuration and electrode materials, and an optimized compatibility need to be considered for multidisciplinary applications. In this review, the unique advantages, key challenges and advanced applications are elucidated in a timely and comprehensive manner. Firstly, the prerequisites for effective integration of the working mechanism and device configuration, as well as the choice of electrode materials are examined. Secondly, the latest advances in the applications of MERABs are discussed, including wearable, self-powered, integrated systems and multisystem conversion. Finally, perspectives on the current challenges and future development are outlined, highlighting the giant leap required from laboratory prototypes to large-scale production and eventual commercialization.

## Highlights

- A timely and updated comprehensive overview focusing on integration of electrochromic aqueous batteries is provided.
- The key prerequisites of integration, basic operating mechanism, and compatibility of the respective components are examined.
- The latest advances and emerging applications are discussed, as well as the future roadmap.

## Graphical abstract

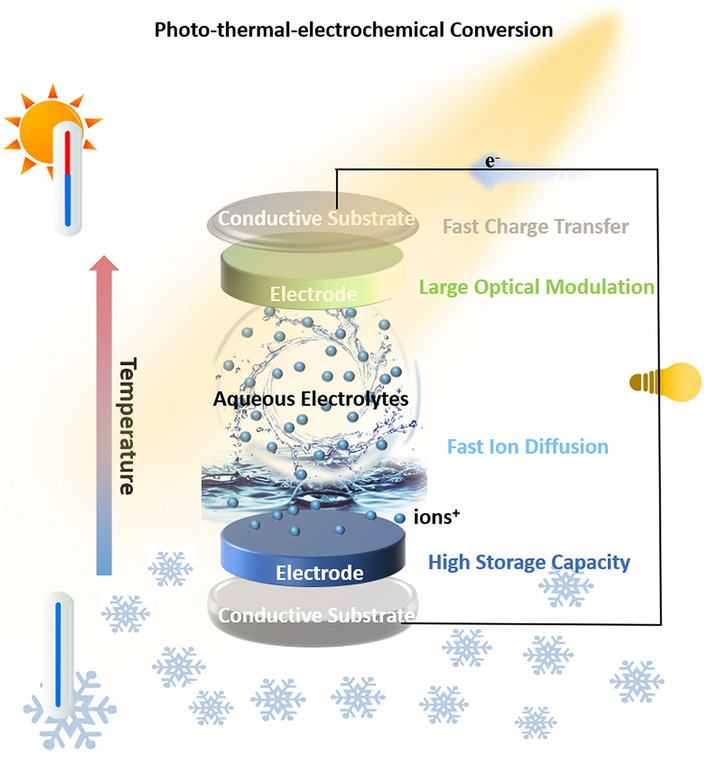

## Introduction

Electrochromism is a light control technology that can be used to autonomously and reversibly change the optical properties of a device (e.g., transmittance, reflectance, and absorbance) based on redox reactions after applying an external voltage bias [1]. Due to the unique feature of vivid switchability in visible color, electrochromic technology has been developed as an ideal energy-saving smart window for decades [2, 3]. Compared with static low-emissivity windows, electrochromic smart windows can be used to effectively reduce energy consumption by at least 10% by managing lighting, heating, ventilation, and air-conditioning (HVAC) [4]. Recently, this color-control technology has been miniaturized and multi-functionalized, which can be expected to be utilized in various new application scenarios including wearable and portable electronics, displays, and energy storage systems [5]. However, the unsatisfactory electrochemical performances, especially slow reaction kinetics, low storage capacity, and poor cycling life, are still the main obstacles for realizing widespread applications [6, 7].

Currently, aqueous batteries with high ionic conductivity and improved overall performance are promising candidates for high power-density devices [8–11]. Compared to toxic and flammable organic electrolytes, aqueous batteries are known to be safe, cost-effective, environmentally friendly, and highly stable devices [12–14]. Therefore, it would be of considerable value to integrate individual advantages into one entity to expand the great application potential of such batteries [15–17]. On the other hand, to meet the ever-increasing demand for energy consumption and the ever-increasing need for various new application scenarios, batteries with additional functionalities are inevitably required. For this purpose, multifunctional electrochromic-induced rechargeable aqueous batteries (MERABs) have been designed to combine electrochromism with aqueous batteries into a single entity, which has been spotlighted in the photo-thermal conversion, thermal-electrochemical systems and energy storage fields [18–20]. MERABs offer additional functionalities of dynamic adjustment of solar light and thermal radiation and spontaneous display of energy levels that conventional aqueous batteries cannot achieve [21, 22].

**Fig. 1:**
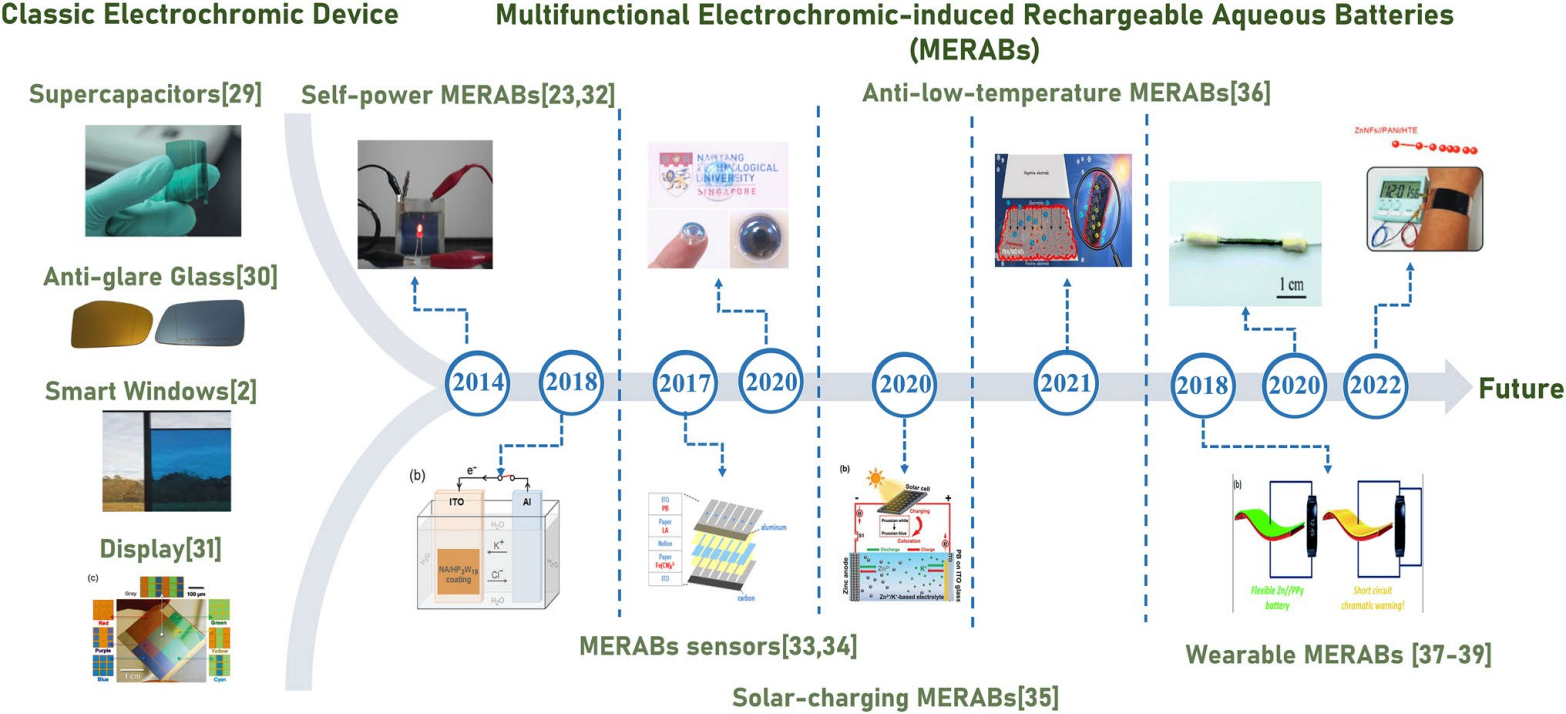
A comprehensive overview of the progress of MERABs: from classic electrochromic device to emerging MERABs. From left to right: reproduced with permission [2]. Copyright 2010, Elsevier. Reproduced with permission [29]. Copyright 2012, Royal Society of Chemistry. Reproduced with permission [30]. Copyright 2015, IOP Science. Reproduced with permission [31]. Copyright 2016 Wiley–VCH. Reproduced with permission [23]. Copyright 2014, Springer Nature. Reproduced with permission [32]. Copyright 2018, Wiley–VCH. Reproduced with permission [33]. Copyright 2017, MDPI. Reproduced with permission [34]. Copyright 2020, Elsevier. Reproduced with permission [35]. Copyright 2020, Wiley–VCH. Reproduced with permission [36]. Copyright 2021, American Chemical Society. Reproduced with permission [37]. Copyright 2018, Royal Society of Chemistry. Reproduced with permission [38]. Copyright 2020, Royal Society of Chemistry. Reproduced with permission [39]. Copyright 2022, Wiley–VCH

Recently, numerous efforts have been devoted to demonstrating prototypes of MERABs. A comprehensive overview of the research progress for MERABs is shown in Fig. 1. Electrochromic technologies were first applied in smart windows and displays, while pseudocapacitive electrochromic windows and self-powered electrochromic batteries were promoted to deeply understand storage mechanisms [23, 24]. To further expand their application scenarios ranging from health checks, harsh environments and Internet of Things (IoT), an integration of multiple disciplines for MERABs has been proposed, including sensors, nanogenerators and wearable electronics. However, the complicated device structure and compatibility of various components lead to several issues of sluggish dynamics, including high interfacial impedance and long ion/electron transport paths [25, 26]. In addition, the low conversion efficiency of any integrated system can also result in a poor energy storage capacity and limited lifespan for MERABs when compared to conventional aqueous batteries [27, 28].

Although there are a couple of reviews on either electrochromic devices or aqueous batteries that are aimed at elucidating the respective challenges and merits [40–42], there is a strong demand for a timely and updated comprehensive overview focusing on the basic design principle and integration of MERABs, especially considering their newly expected applications, together with a critical examination of the perspectives of future research and development. In this review, the key prerequisites of integration, basic operating mechanism, and compatibility of the respective components are discussed. Next, the latest advances and emerging applications of MERABs are discussed ranging from photo-thermal conversion to electrochemistry. Finally, we provide new prospective solutions to the key issues and major challenges in this new and rapidly expanding field, outlining the future development of MERABs and their roadmap.

## Design Principle of MERABs

**Fig. 2:**
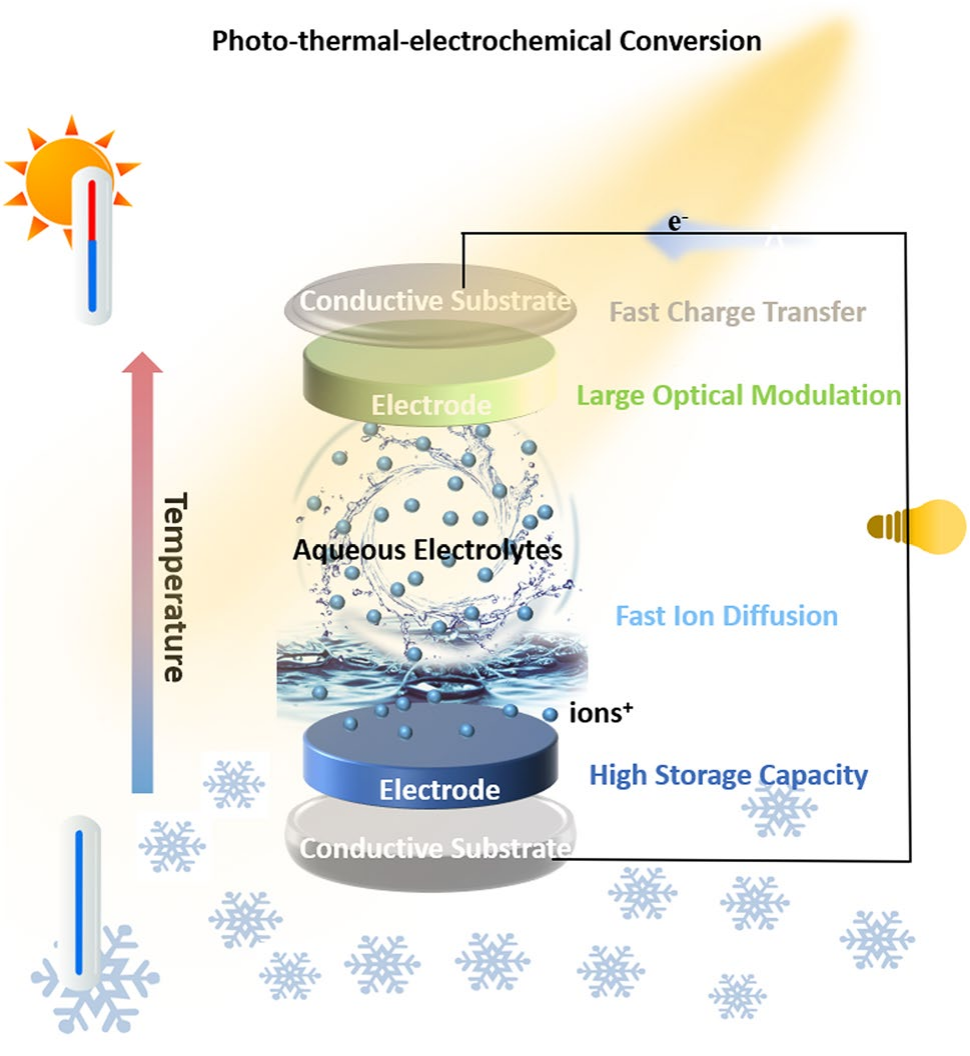
Schematic illustration of the structure and working principle of MERABs

In concept, MERABs are designed to integrate electrochromic and aqueous batteries into one entity with the expectation of executing multiple tasks simultaneously [43]. With this objective, MERABs have brought an emerging and profound understanding of design principles and operating mechanisms, together with the progress in targeted interdisciplinary applications (Fig. 2). Specifically, batteries and electrochromic devices show similar structural characteristics as well as working mechanisms, device configuration, and components, providing sufficient prerequisites for integration [44]. Nonetheless, conventional battery electrode materials with high opacity and poor electrochromic properties limit their applications in MERABs. There are also challenges with the insertion of metal ions, where several key issues of insufficient charge carriers and strong electrostatic interactions need to be further elucidated and understood.

### Potentials and Prerequisites of Integration

**Fig. 3:**
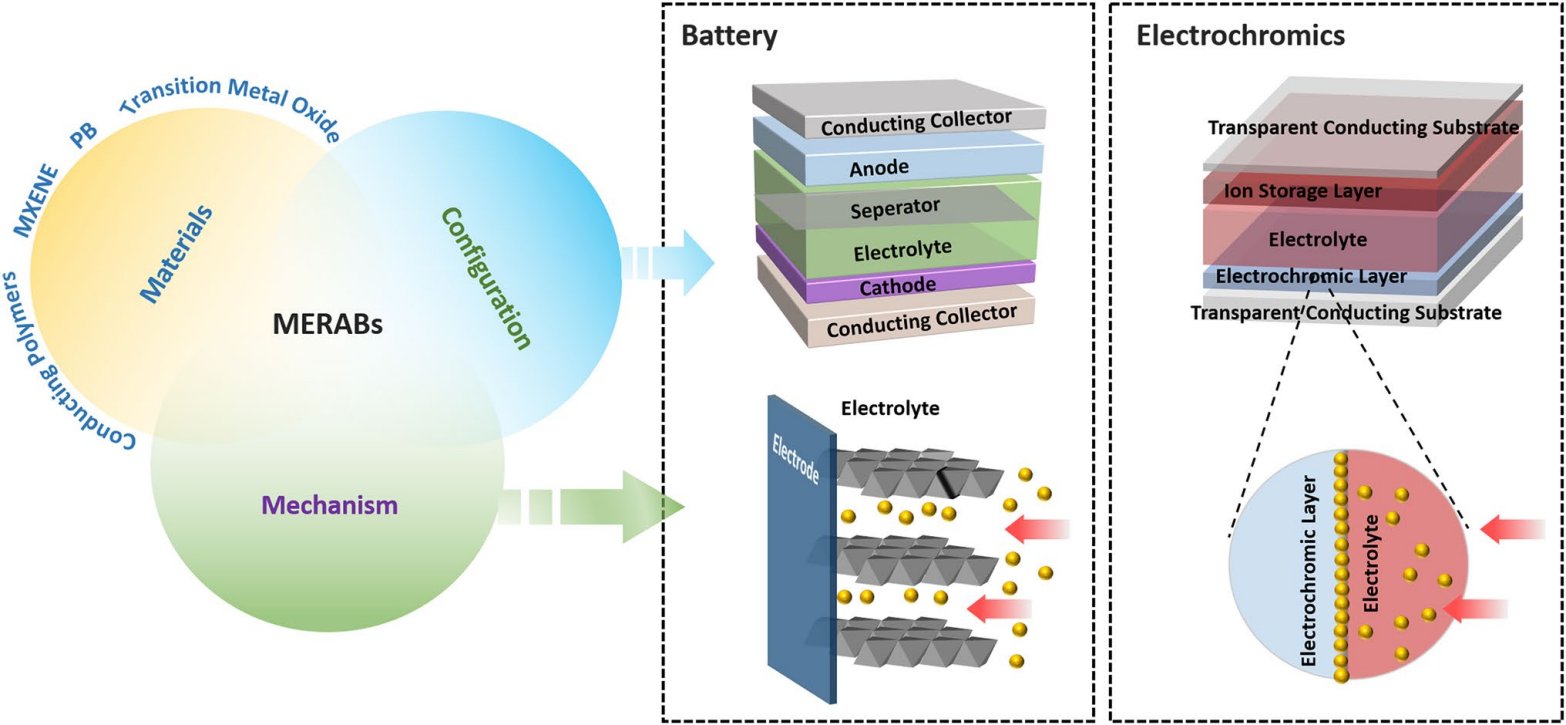
Comparison between the electrochromic device and battery. Schematic illustration of the similarity in electrochromic device and battery in materials, mechanism and configuration

The classical structure of an electrochromic device consists of an electrochromic layer, an electrolyte providing ions, an ion storage layer, and two transparent conducting layers, which are referred to as the cathode, aqueous electrolyte, anode, and two conductive collectors, respectively [45]. The voltage difference between two conducting layers leads to diffusion of ions/electrons toward the electrochromic layer, leading to the corresponding faradic reactions either at the surface or in the bulk. Simultaneously followed by the transportation of counter ions or electrons, a charge-neutral state in the ion storage layer is achieved [46]. This faradic reaction is accompanied by a varying valence or band gap, further resulting in color switching. An ion storage layer for anodic coloration and an electrochromic layer for cathodic coloration affect the optical performance in synergy [47]. It is worth noting that this electrochromic behavior is similar to that of a battery (Fig. 3). Specifically, the faradic reaction of ions occurs in battery electrode materials, enabling the anode to be oxidized and the cathode to be reduced [48]. Essentially, once the connection between the color switching and charge/discharge process is built, it can offer great potential for multifunctional devices, such as displays and indicators.

Given the similarity in the characteristics of the reaction mechanism and device configuration for both electrochromic devices and batteries, it is of great possibility to integrate both into one device. It is noted that the indicators for evaluating the performances of MERABs involve many aspects, such as optical properties, energy storage density, environmental durance, energy conversion efficiency and mechanical properties. Among these properties, the top priority considerations are the optical modulation, cycling life, switching speed, and energy/power density at this stage. The choices of electrode materials and the compatibility of the respective components will largely determine these electrochemical/electrochromic performances.

At present, there are two main categories of electrode materials for MERABs. Battery-type electrochromic materials including Ni(OH)_2_, NiO, V_2_O_5,_ and Prussian blue analogues (PBA) are the most common candidates. The other type of materials is pseudocapacitive-type electrochromic materials, such as WO_3_, TiO_2_ and PEDOT:PSS. On the other hand, some emerging multivalent ions (e.g. Zn^2+^, Ca^2+^, Al^3+^) are known to possess good compatibility in MERAB electrode materials, which further enriches the various choices of integration. Therefore, employing suitable electrode materials and seeking a rational match between the electrode and electrolyte are fundamental and essential prerequisites for successful integrations.

### Electrochromic/Battery Materials (EBMs): From Inorganic Oxides to Conducting Polymers

#### Transition Metal Oxide-based Electrode Materials

**Fig. 4:**
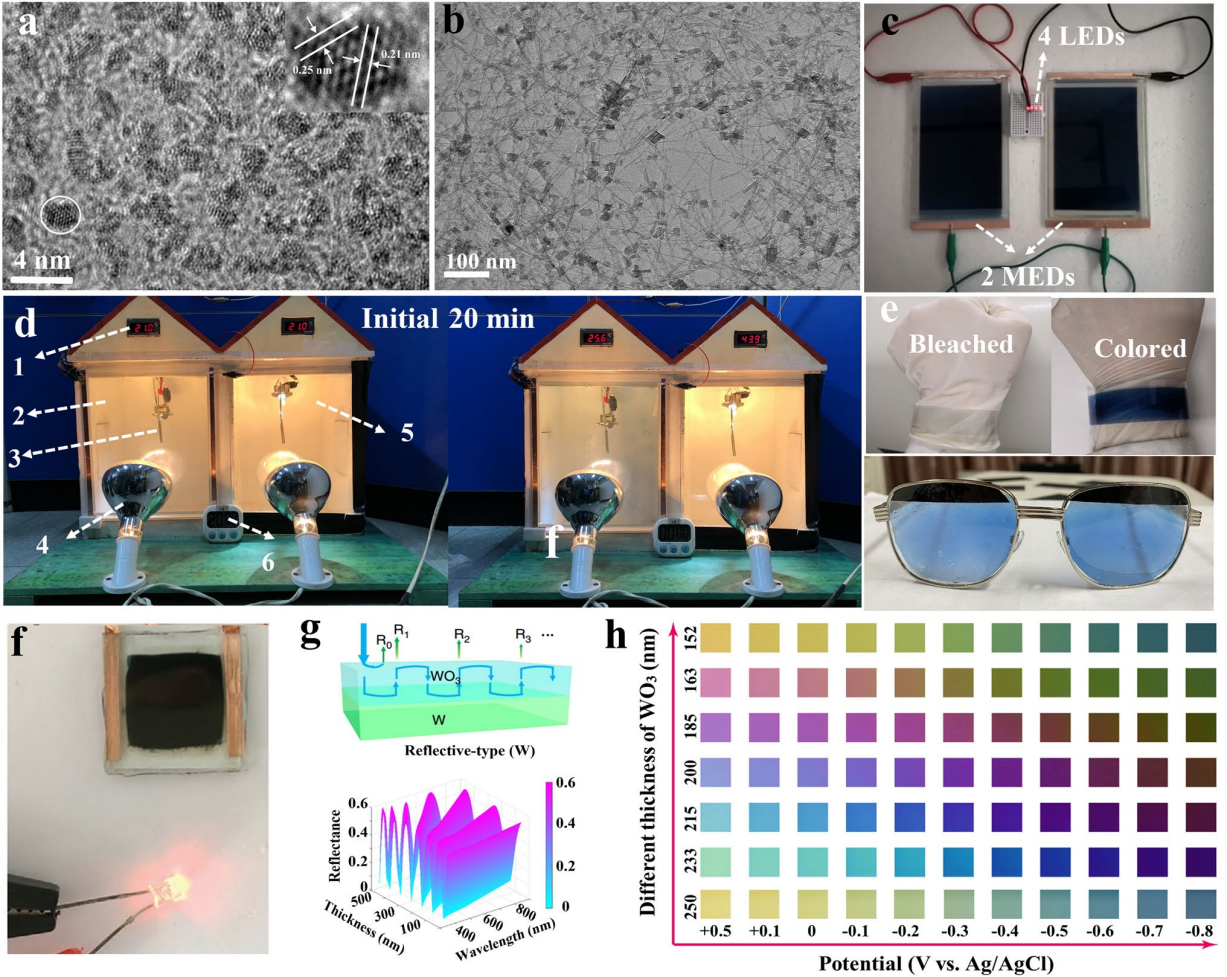
Progress of inorganic oxides in MERABs. **a** Morphology and sizes of WO_3_ quantum-dots [4]. Copyright 2020, Elsevier. **b** Morphology of 3D hierarchical structure composed of W_17_O_47_ NWs knotted together by NaWO_3_ nanoknots. Reproduced with permission [43]. Copyright 2022, Wiley–VCH. **c** Evaluation of the energy storage capacity. Reproduced with permission. **d** Energy-saving simulation in a model house. [21]. Copyright 2021, Elsevier. **e** Wearable wristbands and smart eyeglasses. Reproduced with permission [43]. Copyright 2022, Wiley–VCH. **f** The digital photo of a LED powered by TiO_2-*x*_-based device. Reproduced with permission [51]. Copyright 2020, Wiley–VCH. **g** Novel structure of the F–P type electrochromic device and corresponding simulated transmittance/reflectance spectra. **h** Color gallery achieved by the F–P type electrochromic device. Reproduced with permission [60]. Copyright 2020, Springer Nature. (Color figure online)

Transition metal oxides (e.g. WO_3_, TiO_2_, V_2_O_5_ and NiO) with tunable band gaps can induce strong absorption in both the visible and near-infrared light ranges. Meanwhile, the fast faradic reaction in such oxides can enable a high energy storage capacity for aqueous batteries. However, a slow switching speed and limited color-tuning versatility are two main challenges faced for such EBMs because of the poor electronic conductivity and structural flexibility. Over the past couple of decades, three main strategies have been proposed to address these problems.i*) Size effect: ranging from zero-dimensional dots to one-dimensional nanowires/rods, two-dimensional nanosheets, and three-dimensional (3D) hierarchical structures* [4, 21, 43, 49, 50]. Quantum dots (QDs) with ultra-small sizes can be used to harvest vigorous quantum confinement effect and greatly decrease the interfacial diffusion barrier for ions. A 3D porous network with a large surface area can facilitate ion/charge smooth diffusion, further enhancing the electrochemical performance. Typically, in our previous report, WO_3_ quantum dots with an average size of 3 nm (Fig. 4a) can be used to greatly shorten the diffusion paths and lower the interfacial diffusion energy barrier for the intercalated ions [4]. As a consequence, the prepared WO_3_ QD films exhibit a large optical modulation of 97.8% at 633 nm, a short coloring time of 4.5 s, and an ultra-long cycling life of 10,000 times. In light of this work, we successfully built an integrated system with energy-saving and energy-storage functionalities based on a 3D hierarchical structure consisting of W_17_O_47_ NWs knotted together by NaWO_3_ nanoknots (Fig. 4b) [43]. In particular, this dual-functional device can be used to not only lower the temperature in the model house but also demonstrate the possibility for its use as an effective electric source for LEDs (Fig. 4c, d). This integrated device can also be further applied in various other application scenarios, including smart windows, wearable wristbands, and smart eyeglasses (Fig. 4e). Nanostructures are usually desired to improve electrolyte accessibility and electron-transfer kinetics. However, nanoparticles are normally unstable and easily aggregate when their size is decreased to that of quantum dots. Surface chemistry based on the introduction of multi-functional groups and control of the surface defect distribution is considered to be an effective strategy to maintain stability when designing sub-nanostructures. Specifically, introduction of functional groups changes the surface charge, which favors to anchor nanoparticles in some media or on substrates. Meanwhile, the polarity of functional groups affects the dispersion state of nanoparticles in solvent. Controlling the surface defect distribution can decrease Gibbs free energy to stabilize activity of nanoparticles.ii) *Lattice engineering*. Both doping metal elements and introducing oxygen defects are effective approaches, which aim to improve the concentration of free electrons. A tunable concentration of free electrons can induce localized surface plasmon resonance (LSPR) absorption in the visible (VIS) and the near-infrared (NIR) range. Moreover, the activation energy for ions migrating in the lattice can also be decreased due to the redistribution of electron clouds. Introduction of vacancies increases the electrochemical active sites for inserted ions. Typically, the enhanced diffusion kinetics can result in outstanding optical modulation and energy storage capacity. For example, Lee’s group fabricated a series of TiO_2_-based materials by lattice modification for multifunctional electrochromic systems [51]. Due to a high electron density concentration, the as-prepared TiO_2-x_ films exhibited a high optical modulation (95.5% at 633 nm), satisfying the requirement of personal privacy protection and solar heat reduction. Interestingly, such an integrated device enables a large amount of energy to be recycled during the coloration process. For example, this energy can be used to operate LEDs in the bleaching process (Fig. 4f). Furthermore, a dual-band electrochromic energy storage (DEES) window based on Ta-doped TiO_2_ nanocrystals has been developed, which is capable of independently modulating VIS and NIR transmittance due to localized surface plasma resonance, realizing a simultaneously high charge-storage capacity (466.5 mAh m^−2^ at 150 mA m^−2^) [52]. Notably, thermodynamic favorability is an important prerequisite in designing defect sites, where the chemical environment, the amount of solvent and time should be controlled in synthesis. Typically, thermodynamics calculation of the formation energies in different conditions determines the possibility of the formation of characteristic oxygen-defective structures [53].

(iii) *Compositing: “one-for-two.”* Conducting polymers (e.g., polyaniline, PEDOT-poly(styrenesulfonate) and polyindole) build a “conducting bridge” for electron transportation, and simultaneously enhance the pseudocapacitive capacitance as a result of their excellent ion adsorption ability [54–57]. The switching speed and energy storage capacity can be evenly enhanced by two times [55, 58]. Silver nanowires (AgNWs) and metal grids are also good candidates for their 3D conducting framework [59]. Cai et al. [58] inkjet-printed WO_3_-PEDOT solution onto PEDOT/Ag grid/PET, and observed an ultra-fast speed (t_coloration_ = 2.4 s/t_bleaching_ = 0.8 s). Since PEDOT: PSS acts as a passivation layer to protect Ag from being oxidized, the printed electrochromic films present superior conductivity (0.6 Ω sq^−1^) and flexibility (more than 5000 bending cycles). Notably, asymmetric Fabry–Perot (F–P) nanocavity-type materials have recently attracted much attention for solving the bottleneck of limited color-tuning of inorganic EBMs. Zhao’s group deposited a tungsten (W) layer onto WO_3_ to adjust the reflections at the W and WO_3_ layers (Fig. 4g). A strong interference effect gave rise to multi-color states (the shift of blue–yellow–red) (Fig. 4h). In general, physical mixing and forming chemical bonds are universal compositing methods, especially when combined with high-conductivity materials. Physical mixing is usually through weak interactions such as electrostatic force, hydrogen bond and Van der Waals force. Chemical bonds, including covalent bonds and ionic bonds, are usually strong interactions react. This robust interface structure can effectively improve cycling performance for MERABs. The introduction of a resonant cavity and localized surface plasma resonance endow EBMs with more functionalities. Specifically, a Fabry–Perot cavity has been demonstrated in achieving full-color tunability, which can be used for anti-camouflage textiles. In addition, controlling in the size and shape of metal oxide and metal layers induces tunable LSPR absorption in both the visible and NIR bands, which can adjust photo-thermal conversion. The integration of fundamental mechanisms in interdisciplinary fields, such as optics, electrochemistry and physics, will lead to more applications.

#### Conducting Polymer-based Electrode Materials

Conducting polymers possess high conductivity and good flexibility, where examples are polyaniline (PANI), PEDOT-poly(styrenesulfonate), polythiophenes, and polypyrrole [38, 61–64]. Compared with EBMs with inorganic electrode materials, π-conjugated polymers possess a faster switching speed, higher coloration efficiency, and more color-tuning versatility due to strong π electron delocalization [45].The tinting process for conducting polymers involves ion insertion/extraction, resulting in a change in the band structure (between delocalized π-electron band structures with a LUMO and the original neutral band structures). Thus, these active polymer materials are commonly used for aqueous rechargeable batteries (e.g., Zn^2+^, Al^3+^ and Li^+^). Taking PANI as an example, PANI-based materials generally show various colors of light yellow, green, blue, and purple due to their different redox states [65].With tunable optical and thermal management, it would be valuable to incorporate some of these polymer materials into aqueous rechargeable batteries. For example, Zhi et al. [24] constructed a novel Al//PANI MERAB, delivering a high coloration efficiency of 84 cm^2^ C^−1^, a record-breaking lifespan of 3850 cycles, and superior rate capability. Similarly, PEDOT:PSS, polythiophenes, and polypyrrole are also among the classic electrochromic materials widely applied in displays and smart windows, which are promising candidates for MERABs [66–68].

**Fig. 5:**
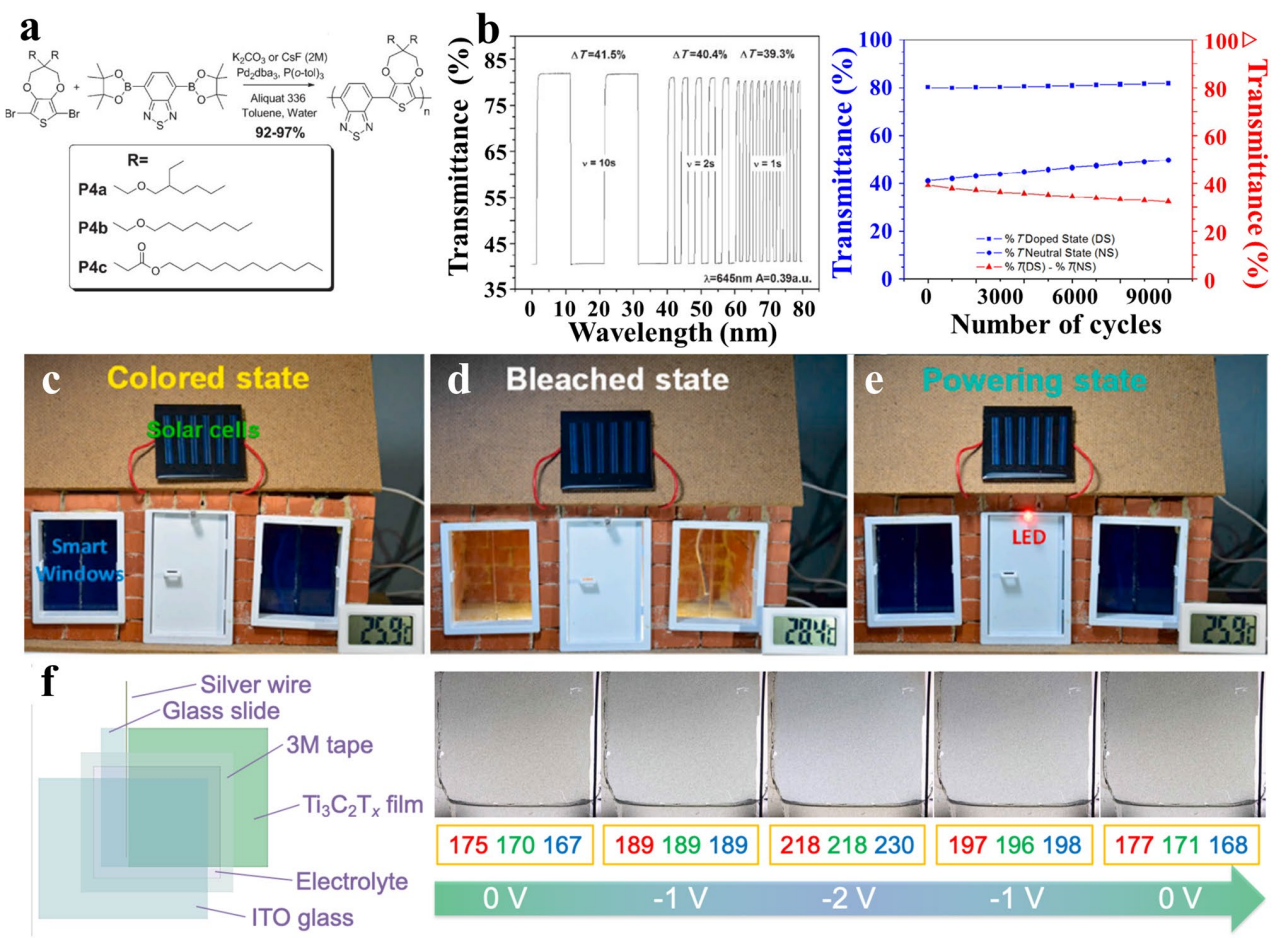
Progress of conducting polymers and other novel electrode materials in MERABs. **a** Structures of DA copolymers. **b** Switching speed and cycling performances. Reproduced with permission [69]. Copyright 2010, Wiley–VCH. **c** Coloring state, **d** bleached state and **e** powering state of the PB-based EES window. Reproduced with permission [73]. Copyright 2020, Elsevier. **f** Schematic illustration of MXene-based device and corresponding digital photos of the as-fabricated device under different potentials. Reproduced with permission [74]. Copyright 2021, Wiley–VCH

However, undesired environmental endurance can lead to poor thermal or photo-stability and a short lifespan. In this case, adopting a donor–acceptor (*D*–*A*) is an effective strategy to lower the band gap. Meanwhile, the activation energy of ions can also be decreased by the guest donor/acceptor, ensuring a low operation voltage and, thereby, reducing the electrochemical damage to the electrode. For example, Reynolds et al. reported a donor–acceptor approach, where two dialkoxy-substituted 3,4-propylenedioxythiophene(ProDOT)-2,1,3-benzothiadiazole(BTD) copolymers gave a long cycle life (over 10,000 times) and subsecond response time (less than 1 s) (Fig. 5a, b) [69]. Conducting polymers possess a unique feature of wide tunability for the amount of EC molecules lacking in transition metal oxides. More specifically, conducting polymers can act as redox species that are soluble in the electrolyte to form free radicals. This novel and simplified design named an “all-in-one” architecture. Typically, “all-in-one” architecture is fabricated by directly introducing ion-soluble chromophores and counter redox materials into the electrolyte to form one single layer [70–72]. In comparison with conventional five-layered devices, such simplified devices are easy to fabricate without any high-temperature treatment and vacuum processing. In addition, interface engineering between electrode layers and electrolyte layers is not necessary, which offers a high degree of coloration.

#### Other Novel Electrode Materials

Prussian blue analogs (PBAs) are among the representative cathode materials for both zinc aqueous batteries and anodic electrochromic materials in electrochromic devices, as their open 3D frameworks can provide spacious channels for charge transportation [75, 76]. Mai et al. [73] developed a bi-functional PB-based electrochromic energy storage (EES) window. The integrated EES window was shown to exhibit three different color states of transparency (PW), dark blue (PB), and green yellow (PY), showing the potential of being able to effectively control indoor light and temperature during the daytime (Fig. 5c, d). A fully charged EES window was demonstrated in powering red LEDs at night, thereby realizing photo-thermal-electrochemical conversion (Fig. 5e). In addition, PBAs also show much promise toward storing various ions (e.g., Na^+^, K^+^, Mg^2+^ and NH_4_^+^) for aqueous batteries. Furthermore, a mild PBA synthesis route can be easily scaled up for industry when compared with that for transition metal oxide and conducting polymer materials [77]. However, two main issues of interstitial water and vacancies need to be addressed, where interstitial water can occupy the active sites for ions and vacancies can induce a distortion in the lattice structure.

Transition metal carbides, nitrides, and carbonitrides (MXenes) have been studied for rechargeable batteries due to their high conductivity, redox properties, and large inter-layer spaces. Notably, the abundant functional surface groups (Ti–O or Ti–OH) of MXenes show pseudocapacitive behaviors, inducing the reversible change of Ti valence. Moreover, metal-like free electron of the MXenes can be tunned especially when potentials applied, which can strongly induce the surface plasmon. Therefore, deep investigations of their optoelectronic properties and tunable surface groups can provide new insights into their applications in electrochromic devices. For example, Gogotsi et al. [74, 78] have demonstrated that Ti_3_C_2_T_*x*_ can function as both a conductive material and electrochromic material through the reversible redox of titanium by (de) protonation of oxide-like surface functionalities (=O and –OH) (Fig. 5f). This piece of work expands optoelectronic and photonic applications for MXenes, which are emerging as a new class of electrochromic materials for MERABs.

**Fig. 6:**
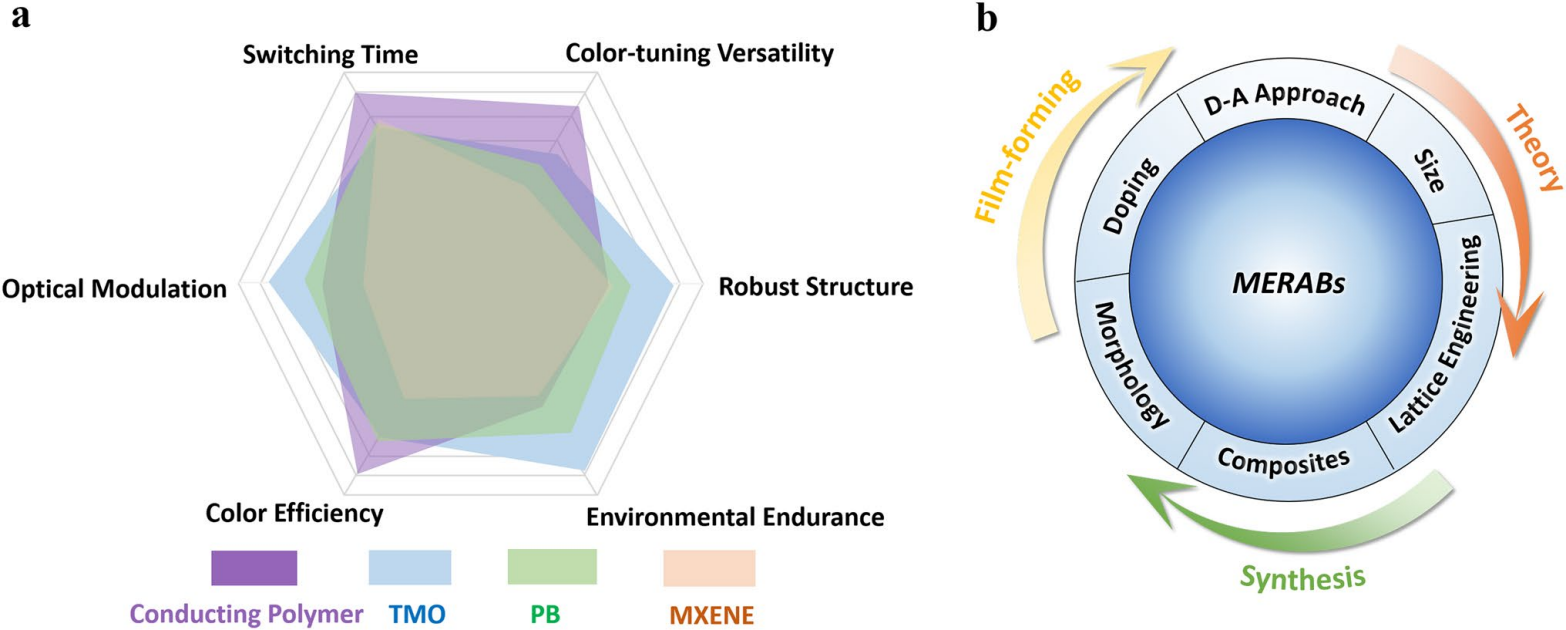
Comparison of typical MERABs materials and main modified strategies. **a** Summary of the typical characteristics of different MERABs materials. **b** Schematic diagram showing the main strategies for improving electrochemical and electrochromic performances of MERABs

Generally, MERAB materials are still lacking and are mainly limited to electrochromic materials in current research. As shown in Fig. 6, each of these materials has its own pros and cons. Inorganic EBMs have robust lattice structures but show relatively slow coloring time and poor color-tuning ability. Conducting polymer EBMs offer a sub-second switching time, but are vulnerable to the external environment, such as oxygen, moisture, and light. However, it is worth also noted that some of the emerging EBMs, such as MXenes and MOFs, cannot effectively modulate the transmittance. Great efforts should be made to compensate for their disadvantages. Even though some remarkable progress has been achieved, it is still worth noting that the contradiction between the large coloration efficiency (CE) and high energy/power density needs to be further balanced. Ideally, a smaller voltage is required to induce a larger optical transmittance for an electrochromic device. Instead, a high voltage is needed to realize a higher energy/power density for a battery. Additionally, a controllable thickness for MERABs can greatly affect the comprehensive performance. Thin films can lead to low capacitance for aqueous batteries, and thick films can result in a slow switching speed for the transmittance of electrochromic devices. For applications requiring a fast switching time and colorfulness, such as smart glass or displays, organic EBMs would be among the choices. In contrast, if the device function is focused on supplying power and energy sources, inorganic EBMs with battery behaviors would be preferred, such as PBA, NiO, and V_2_O_5_. Therefore, there are different focuses on materials properties and processing parameters for different applications.

### Compatible Aqueous Electrolytes: From Mature Monovalent to Multivalent-Ions

Reaction kinetics in electrolytes play an essential role in electrochemical and electrochromic performance. The compatibility of inserted ions with host materials can significantly affect the ion diffusion barrier and ion-trapped depth [12]. Specifically, the intrinsic properties of inserted ions, including the electrostatic interactions, polarity, and redox potential, need to be considered comprehensively and critically. Since Deb [79, 80] first reported the electrochromic behavior of tungsten oxide in 1969, H^+^ (H_2_SO_4_), Li^+^ (LiCl, LiClO_4_), and K^+^ (KCl) electrolytes have been widely investigated [81–84]. In particular, Li^+^ ion-based electrolytes, as the most mature system, have been commercialized in the fields of both batteries and electrochromic windows. However, each of them has its own limitations as far as MERABs are concerned: (i) the relatively high cost of lithium-ion electrolytes; (ii) the strong corrosion behavior of H^+^; and (iii) the limited choices of electrode materials for K^+^. Therefore, searching for electrolytes with low cost, environmental friendliness, and good compatibility is important. Recently, both alternative monovalent ions (e.g., Na^+^) and multivalent ions with small ionic radius have been studied. In this section, aqueous electrolytes are divided into monovalent-ion types and multivalent-ion types for discussion.

#### Monovalent-Ion Aqueous Electrolytes

H^+^, Li^+^, Na^+^, and K^+^ ions as inserted ions are commonly used for both electrochromic and battery systems. Li^+^-based energy storage systems have been widely explored due to their advantages of fast kinetics and high electrochemical performance. Li^+^ ions can well match most electrochromic-induced aqueous battery materials. In this case, pseudocapacitive smart windows, multicolor electrochromic displays, and energy-saving electrochemical devices have emerged in recent years [83, 85]. Meanwhile, great progress in the understanding of lithiation mechanisms has also been achieved. It is commonly believed that a phase transition is responsible for Li^+^ storage in WO_3_ lattices [86–88]. Li^+^ ions will first diffuse from the triangular cavities to the quadrilateral cavities and then are located in the hexagonal cavities in WO_3_ to form Li_2_WO_4_ [89]. Even though the structure of h-WO_3_ can be well maintained during the lithiation process, repeated electrochemical intercalation/deintercalation processes can also gradually result in optical degradation and irreversible color change. Considering the importance of the depths or sites for ion trapping, Granqvist et al. proposed an "ion-trapping" mechanism to explain this phenomenon [90]. Specifically, shallow traps (with low-energy barriers) and deep traps (with high-energy barriers) involve different processes. Shallow trapping can deliver a fast ion deintercalation process and ensure the possibility of long-term electrochemical cycling. In contrast, it is much more difficult for those ions to be trapped at deep sites. To further address this issue, Wen et al. [91] proposed a galvanostatic treatment strategy to eliminate ion-trapping-induced degradation to rescue ions trapped in deep sites and revive the original optical and electrochemical performances.

Compared with the abovementioned Li^+^, H^+^ has a small ionic radius of 0.29 Å, but its strong corrosion behavior can result in serious degradation of the electrode, limiting its practical application. For example, Gao et al. systematically investigated the electronic and optical properties, and the ion diffusion behavior of WO_3_ with different electrolytes containing H^+^, Li^+^, and Na^+^ by Ab-initio calculations [92]. They reported that the diffusion energy barrier for H^+^ is smaller than that for Li^+^ and Na^+^ (H^+^ < Li^+^ < Na^+^). However, H^+^ prefers to occupy the interstitial site in the W–O layer instead of the *A* site favored by Li^+^ or Na^+^ in the ABO_3_ structure, which will induce a larger lattice distortion for WO_3_ and induce serious structural degradation. Instead, considering their abundant source and high degree of safety, Na^+^-based aqueous electrolytes are attracting more attention. However, the ongoing development of Na^+^-based MERABs is still in an embryonic stage due to the sluggish diffusion kinetics and the limited sites for Na^+^ occupation considering the large ionic radius of Na^+^ (1.02 Å) [93]. In addition, conventional Na metal anodes are very unstable, which are sensitive to O_2_ and H_2_O. The electrochromic /electrochemical performance is far from the levels required for practical applications. A similar dilemma is also faced by the K^+^ ions due to the much larger ionic radius of 1.33 Å. Even though graphene/PB multifunctional electrodes have been proven in transparent aqueous K^+^ ion batteries/electrochromic devices, slow charge transport kinetics and irreversible structural degradation during K^+^ insertion/extraction cannot be ignored [94]. Notably, K^+^ in alkaline solution has been investigated for decades, initially in batteries and later also for electrochromics. For example, KOH is widely used for NiO host materials [95]. Numerous works show an excellent adsorption in visible wavelength. However, alkaline electrolyte is generally not suitable for other host materials; therefore, controlling of pH value is important in optimizing the electrochromic performances.

To improve the diffusion kinetics of ions with larger ionic radius, atomic-level lattice engineering has been explored as an effective approach to manipulate the internal space and lattice defects in electrode materials. Yao et al. [96] reported that the ion diffusivity can be enhanced by two orders of magnitude after increasing the interlayer spacing of MoS_2_ to 1.45 nm via introducing poly(ethylene oxide) between layers. Nonmetal ammonium ion (NH_4_^+^) with a special isotropic tetrahedral structure is another alternative candidate [97] in terms of the abundant source from plant fertilizers [98]. Similar to the progress for Na^+^/K^+^ ion research, the exploration of effective host materials for NH_4_^+^ ions is still at the stage of preliminary investigations and the acknowledged storage and degradation mechanism has not been well established.

#### Multivalent-Ion Aqueous Electrolytes

**Fig. 7:**
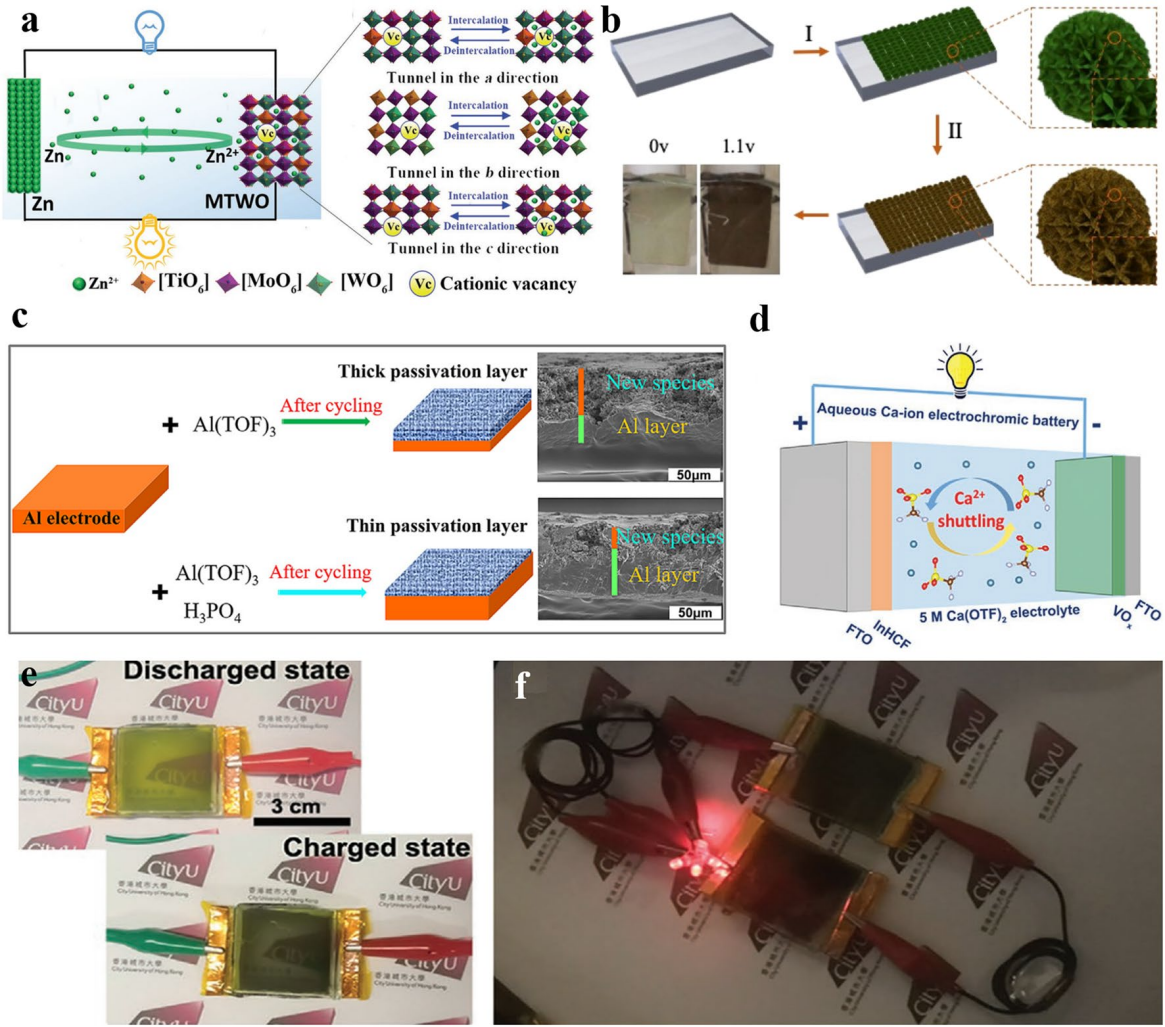
Progress of multivalent-ion aqueous electrolytes in MERABs. **a** Schematic illustration of Zn^2+^-based MERABs. Reproduced with permission [101]. Copyright 2019, Wiley–VCH. **b** Preparation procedures of the Ni-MOF open framework. Reproduced with permission [102]. Copyright 2020, American Chemical Society. **c** Schematic showing the passivation film formation. Reproduced with permission [24]. Copyright 2021, Elsevier. **d** Schematic illustration of the Ca^2+^-based MERABs system. Demonstrations of **e** color modulation, and **f** powering of Ca^2+^-based MERABs. Reproduced with permission [105]. Copyright 2021, Wiley–VCH

Emerging multivalent ions, such as Zn^2+^, Ca^2+^, and Al^3+^, have also been studied for MERABs because of the rich electron transfer which can offer a high volumetric energy density together with excellent optical modulation [18, 99, 100]. Given the low redox potential (− 0.76 V vs. SHE) and high gravimetric capacity (820 mAh g^−1^) of Zn^2+^, Elezzabi et al. [35, 101] successfully carried out a series of studies for zinc-based MERABs, including self-recharged MERABs and solar-charging MERABs, which show great potential to reduce energy consumption in low-carbon society. To suppress the stronger electrostatic interactions between multivalent ions and the host lattice, Ti^2+^ has been introduced into tungsten molybdenum oxide to increase the number of electrochemical sites, which delivers a large areal capacity of 260 mAh m^−2^ and high optical contrast (76%), as shown in Fig. 7a [101]. Meanwhile, structure construction offers another approach for manipulating fast Zn^2+^ diffusion kinetics. A conductive metal–organic framework (MOF) layer has been constructed on a NiO@C electrode which has been adopted by Jia et al. [102] to guide the uniform Zn^2+^ diffusion via the uniform channels (Fig. 7b). As a result, the designed NiO@C electrode showed a fast coloring time of 8.5 s, and realized a zero transmission in the whole visible light range. In general, Zn foil or mesh was employed as the anode. However, most studies have investigated the reaction mechanism and synthesis method of cathodes, but the related development for modifying Zn anodes is rather limited. It is noted that Zn dendrite behavior and interfacial side reactions need to be urgently addressed. Similar to Zn^2+^, Al^3+^ ions not only possess a small ionic radius (0.53 Å), but also can support three-electron redox reactions. This means that the involved electrons are equivalent to three times by monovalent ions. However, Al metal in an aqueous electrolyte undergoes severe and passivates oxide film formation [103]. A strong electrostatic interaction between the host material and Al^3+^ ions also often leads to lattice expansion and inferior reversibility. To address these issues, Zhi et al. [24] introduced H_3_PO_4_ additives into an Al(TOF)_3_-based electrolyte to form complex cation Al(H_2_PO_4_^−^)_*x*_(TOF^−^)_*y*_^+^(H_2_O)_*n*_ solvation complexes (Fig. 7c), which can be used to effectively alleviate the formation of a passivation layer and reduce the strong charge densities of Al^3+^. Unlike the wide research for Zn^2+^ and Al^3+^, the development of Ca^2+^-based MERABs is rather limited even though Ca^2+^ ions possess comparably low polarization with Li^+^ when compared with the multivalent ions of Zn^2+^, Mg^2+^, and Al^3+^. Furthermore, the narrow electrochemical window and large radius of hydrated Ca^2+^ ions are also problematic issues [104]. Lee et al. [105] successfully constructed a MERAB prototype that involved the integration of multicolor (greenish-yellow-to-black) electrochromism and aqueous Ca-ion batteries as shown in Fig. 7d–f. A water-in-salt (WIS) Ca(OTF)_2_ electrolyte was employed to promote Ca^2+^ desolvation and widen the electrochemical window, where cation–anion pairs are increased and the coordinated water molecules are reduced in hydrated Ca^2+^ ions. However, the exact mechanism of Ca^2+^ ions storage has to be properly studied for better understanding.

**Fig. 8:**
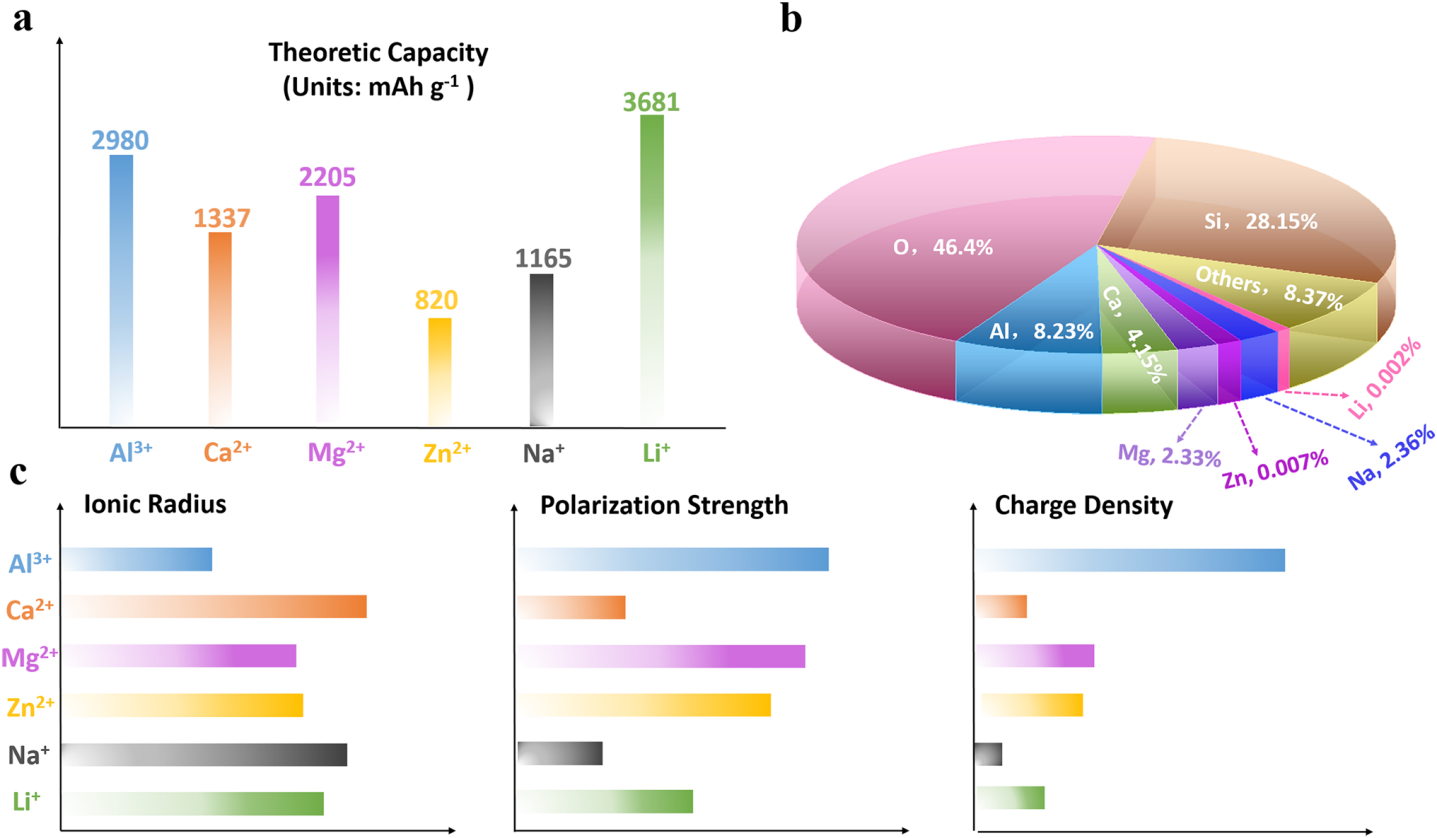
The intrinsic properties of different inserted ions [110, 111]. **a** Theoretical capacity. **b** Content of elements in earth curst. **c** Comparison of ionic radius, charge density, and polarization of the common inserted ions

Overall, Li^+^-based electrolytes are among the most common and mature systems for MERABs. With the drawbacks of relative scarcity, attention has been moved to other alternative monovalent-ion and multivalent-ion systems. As summarized in Fig. 8a, b, Na^+^ is a promising next-generation candidate in the monovalent-ions system due to its high theoretical capacity and low cost. Atomic-level lattice engineering is an effective approach for addressing the slow diffusion kinetics of Na^+^ to a certain degree. For those ions with multivalent states, Zn^2+^ and Al^3+^ are preferred due to their relatively smaller ionic radius and abundant element resources. However, strong polarization is the main obstacle in comparison with monovalent ions (Fig. 8c). Through carefully designing the 3D conductivity framework with uniform ion transfer channels, strong polarization can be weakened, and the ion diffusion ability will also be improved. In addition, the well-matching of the electrode and electrolyte is also a key factor toward realizing high device performance. Although the degradation mechanism for the metal anode is often ignored, one should focus on solving the metallic dendrite and side reaction in parallel with the cathode materials. At the same time, a deep understanding of the reaction mechanism is also very important in searching for novel cathode materials. Last but not least, easy encapsulation and chemical stability are prime considerations for practical applications. Liquid electrolytes suffer some drawbacks of bubbles generation and leakage risks, while all-solid-state electrolytes have a rather low ionic conductivity. Instead, semi-solid gel electrolytes take advantages of high ionic conductivity, eco-friendliness, and tailorability. Particularly, hydrogel electrolytes as typical semi-solid electrolytes, consist of elastic crosslinked hydrated polymer chains with high content of water. Efforts on polymer chemistry and polymer engineering enable the hydrogel materials with great designability and tunability [106]. These features endow hydrogel electrolytes functionalities of stretchability, self-healing ability, and temperature adaptability, which are appealing for MERABs in photo-thermal systems and wearable electronics [107, 108]. Notably, different ions (e.g., Li^+^, Na^+^ and Zn^2+^) have different interactions with hydrogels; therefore, employing suitable types of polymer and electrolytic salts, together with reasonable theoretical interpretation, is highlighted for MERABs [109].

### Conducting Substrates: From Basic Demand to Smart Functionality

**Fig. 9:**
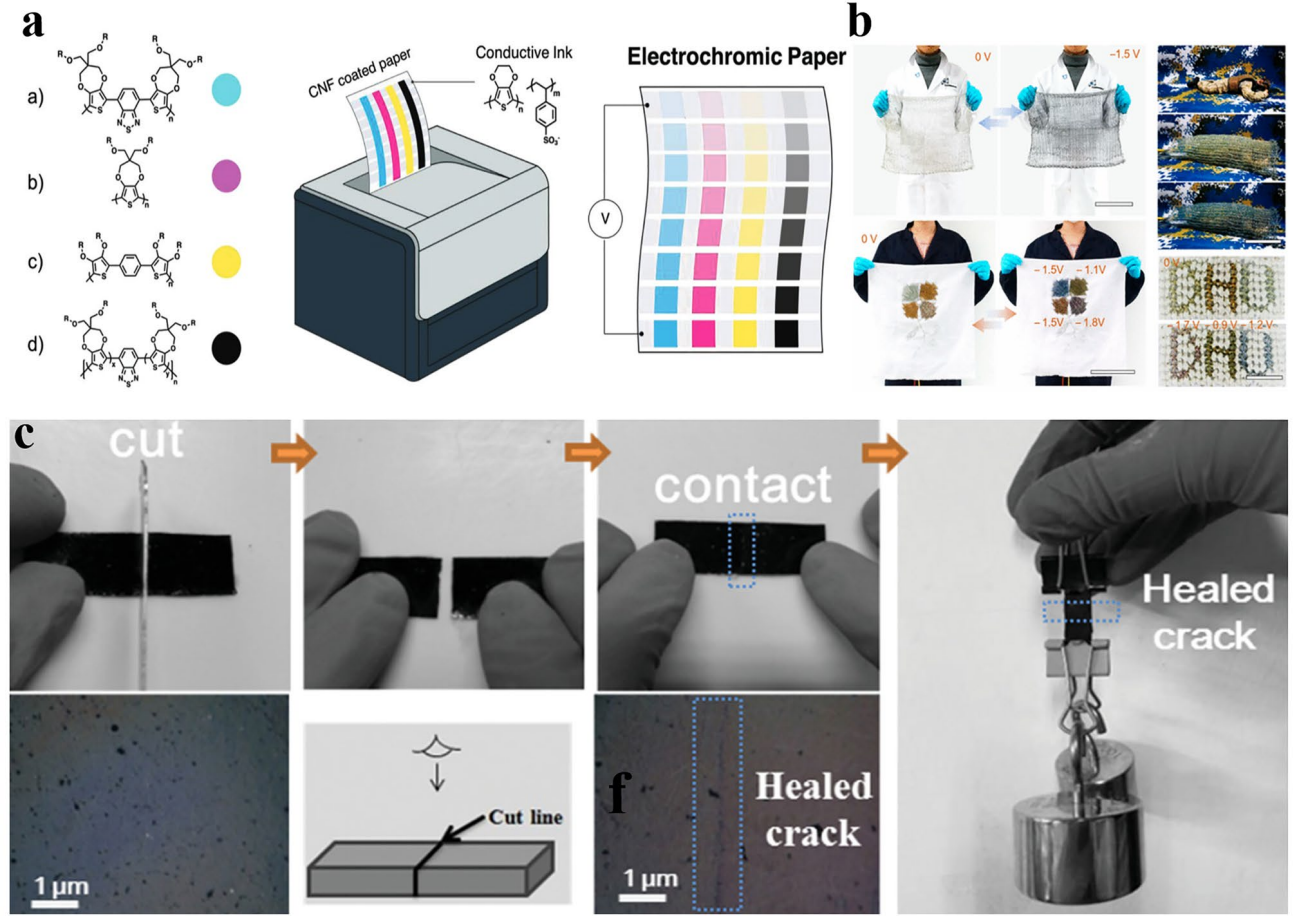
Progress of conducting substrates in MERABs. **a** Electrochromic paper-based displays. Reproduced with permission [114]. Copyright 2019, Wiley–VCH. **b** Anti-camouflage clothing. Reproduced with permission [115]. Copyright 2020, American Chemical Society. **c** Digital photo of the self-reparability of reinforced self-healed polymer and the corresponding SEM images of the surface of reinforced self-healed polymer. Reproduced with permission [116]. Copyright 2019, Wiley–VCH

The general requirement for substrates in MERABs is similar to that in conventional batteries, except for the unique transparency of MERABs. To meet the rapid emergence of the Internet of Things (IoT) and new wearable electronics, other key features, including foldability, stretchability, and self-healing capability are also required for MERABs. To date, poly(ethylene terephthalate) (PET), polyurethane (PU), an poly(dimethylsiloxane) (PDMS), nanocellulose-based and fiber-based materials have been reported as substrates for MERABs [112]. Carbon-based (carbon nanotubes and graphene), metal nanowires/grids, conducting polymers, and MXene are promising conductive materials, that can be easily incorporated into substrates [113]. As mentioned above, foldability and stretchability are the basic features of flexible electronics to ensure the device can maintain the electrochemical performances upon repeated twisting and stretching. With the advantages of being bioderived and biodegradable, nanocellulose-coated and fiber-based substrates possess great opportunities for next-generation flexible electronics such as paper-based displays [114] and anti-camouflage clothing [115] (Fig. 9a, b). In addition to the basic features/requirements of the substrate in flexible electronics, another interesting feature is self-healing, which can recover the primary functionalities after unforeseen damages [116, 117]. As depicted in Fig. 9a, c healable thermoplastic elastomer material can recuperate enormous extensibility. The high repeatability of breaking and healing can be realized via the formation of hydrogen bonds. These additional smart functionalities of substrates further offer the possibility for the comprehensive applications of MERABs.

## Recent Advances in MERABs

### From Rigid to Wearable MERABs

**Fig. 10:**
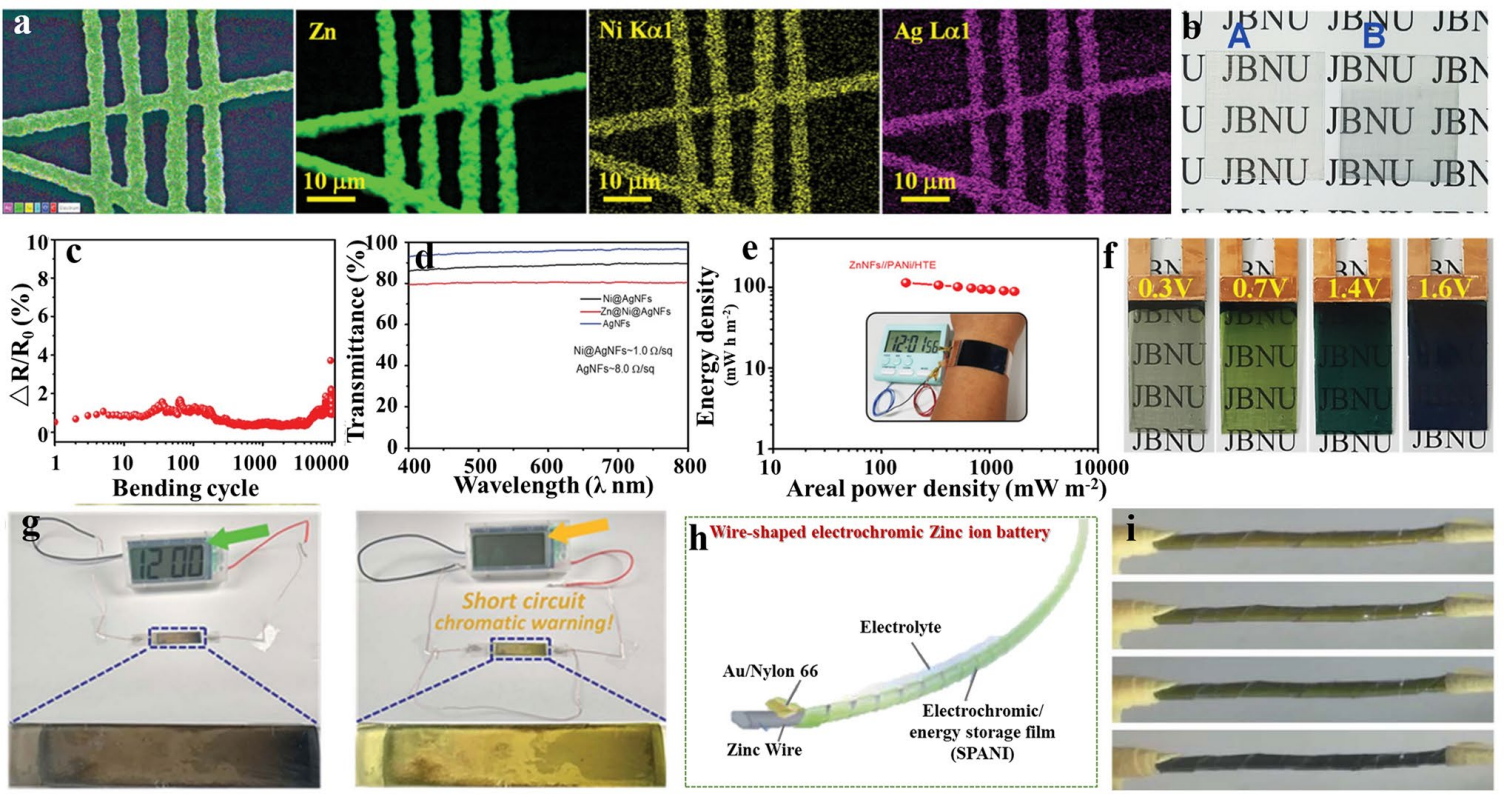
Advances in wearable MERABs. **a** EDAX mapping images of the Zn@Ni@AgNFs network. **b** Digital photographs of the Zn@Ni@AgNFs electrode. **c** Bending test of the Zn@Ni@AgNFs electrode. **d** Optical spectra of the Zn@Ni@AgNFs electrode. **e** Ragone plot of the MERABs using Zn@Ni@AgNFs electrode. The inset shows the wearable MERAB powering an alarm clock. **f** Demonstration of color-tuning ability. Reproduced with permission [39]. Copyright 2022, Wiley–VCH. **g** The prototype with short circuit chromatic waring functions. Reproduced with permission [37]. Copyright 2018, Royal Society of Chemistry. **h** Schematic illustration of the structure of the wire-shaped zinc-ion battery. **i** Digital photos of the coloring and bleached states of the wire-shaped zinc-ion battery. Reproduced with permission [38]. Copyright 2020, Royal Society of Chemistry. (Color figure online)

Much progress has been witnessed on flexible conducting substrates, which has promoted the rapid development of MERABs with satisfied mechanical endurance. Together with aqueous batteries and electrochromic devices, the integrated MERABs are one of the potential next-generation electronics in smart textiles, flexible displays, and daily health checks [118–121]. Lee et al. proposed transparent wearable zinc-based MERABs with Zn@Ni@AgNFs core–shell materials as anode to avoid the opaque and rigidity of the Zn metal anode [39] (Fig. 10a). The Zn@Ni@AgNFs anode displayed high optical transparency (~ 80% at 550 nm) and good mechanical flexibility (only a 5% increase in resistance after 10,000 bending times) (Fig. 10b–d). Moreover, a suitable match between the electrochromic electrode and electrolyte is also important for achieving both high electrochemical and electrochromic performance in MERABs. Lee et al. proposed to electrodeposit PANI on hybrid PEDOT:PSS/AgNFs to assemble a multicolor cathode and matched with PVA–ZnCl_2_ gel electrolyte to suppress the degradation after stretching and bending. This prototype MERAB demonstrated a high volumetric energy density (378.8 Wh m^−3^ at a power density of 562.7 W m^−3^) and a large optical contrast (50%), as well as a robust electrochemical and electrochromic stability (retention of initial performance after repeated mechanical deformations) (Fig. 10e). More interestingly, the charge states are visibly marked by various controllable colors, indicating that the energy density state of the MERAB can be visually monitored in real time. For example, the MERAB presented the transparent, light green, dark bluish green and dark bluish-violet appearances under the voltages of 0.3, 0.7, 1.4 and 1.6 V, respectively (Fig. 10f). Given the color-changing features, the chromatic warning function of the short circuit had also been realized in the integrated MERABs by Huang et al. [37] As shown in Fig. 10g, the battery appears black when it is working, and it turns bright yellow at once and the clock turns off under short-circuit conditions. In addition to the abovementioned functionalities, Jia et al. [38] reported a wire-shaped MERAB, which was assembled with a self-doped PANI cathode and Zn wires intertwined by Au slender strip as presented in Fig. 10h, i. Typically, this multi-color MERAB showed a high specific capacity of 23.2 mAh cm^−3^ at 0.1 A cm^−3^, which can easily power an electronic clock even under various deformed states. In addition, this design is promising for being woven into smart clothing, for applications in anti-camouflage and emergency power supply in outdoor activities.

In general, both aqueous batteries and electrochromic devices have been explored for applications in wearable, health care and implantable devices [122, 123]. Nevertheless, the integrated MERABs are still at the initial stage of development due to the lack of high-performance electrode materials and substrates. Searching for novel electrode materials and substrates with the required mechanical properties is imperative for next-generation wearable MERABs, where carbon-based materials, MXenes and conducting polymers could be the best candidates. In addition, the conventional “sandwich” structure for wearable MERABs is not applicable in terms of their thick and heavy configuration. “All-in-one” architecture might be a promising choice, which effectively decreases the thickness of the whole device. Moreover, developing a desired electrochemical system, including an ideal match of electrodes and electrolytes, is crucial to balance the trade-off between the large coloration efficiency and high energy/power density.

### From External Energy Supply to Self-powered MERABs

**Fig. 11:**
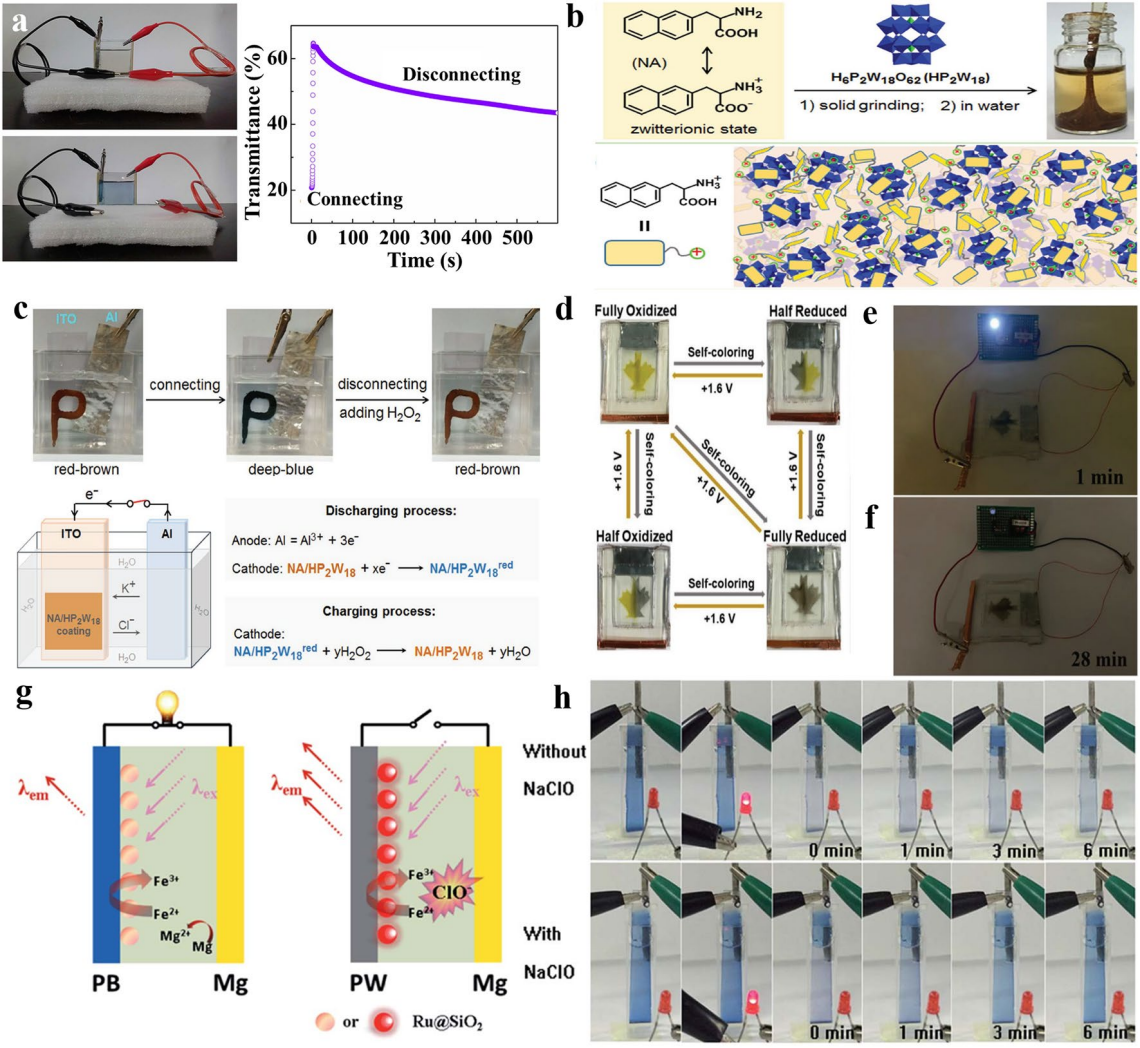
Advances in self-powered MERABs. **a** Demonstration of the color restoration process without external input power, and the corresponding *in-situ* transmittance spectra of self-powered MERABs with connecting and disconnecting situation. Reproduced with permission [23]. Copyright 2014, Springer Nature. **b** Chemical structures of NA and HP_2_W_18_ and the corresponding schematic packing model of the NA/HP_2_W_18_ adhesive. **c** Demonstration of the NA@HP_2_W_18_@Al device with reversible color switching, and the corresponding schematic illustration of the working mechanism. Reproduced with permission [32]. Copyright 2018, Wiley–VCH. **d** Digital photographs of the self-powered display with color-tuning ability. **e–f** Digital photographs of 0.5 V LED powered by self-powered display. Reproduced with permission [125]. Copyright 2019, Wiley–VCH. **g** Scheme of self-powered Mg/PB MERABs. **h** Digital photographs of self-powered MERABs with/without NaClO_4_. Reproduced with permission [126]. Copyright 2016, Royal Society of Chemistry. (Color figure online)

Most MERABs require an external power source, which will increase the extra energy consumption and complexity of the configuration. Self-powered MERABs would be an effective strategy to address these issues and have received considerable attention. In general, the self-powered MERABs can be mainly divided into two categories: (i) integration of a harvesting system, such as triboelectric nanogenerators (TENGs), solar cells and sensors, into MERABs; (ii) a single entity composed of self-powered MERABs. The former strategy shows the advantage of being highly controllable, but the efficiency is quite low due to the complicated configuration, which would be discussed in the following section. In comparison, the latter strategy is more effective and can preserve the whole entity of MERABs, which only utilize the external environment (e.g., oxygen, light, or introducing additives) and intrinsic properties (e.g., redox potential difference) to realize self-supplied power. As shown in Fig. 11a, Wang et al. employed Al foil, Prussian blue and KCl solution as the anode, cathode and electrolyte, respectively, to fabricate self-powered MERABs [23]. The strong reduction capability of Al induces a large potential difference between the Al anode and PB cathode, providing enough driving voltage to bleach/discharge the MERAB itself. The bleached MEARB spontaneously switches back to its original blue color after the disconnection of electrodes and simultaneously lights up the LED. This self-recharge phenomenon is attributed to the oxidation of Fe(II) to Fe(III) to form PB via oxygen in air. Notably, oxygen in MERAB is only involved in the self-charging/coloring process, which is different from that of metal-O_2_ batteries. However, given the low specific capacity (63.6 mAh g^−1^) and long self-charging time (12 h), introducing H_2_O_2_ should be an effective approach to accelerate the oxidation reaction. Zhao et al. [124] reported that the potential difference between WO_3_/Al foil and electrochemical performance can be significantly enhanced in the presence of H_2_O_2_, and such MERAB can deliver a high discharge capacity of 429 mAh g^−1^ and a fast self-charging speed (8 s). Li et al. [32] also reported a novel hetero-polyacids (HPAs)/water-immiscible amino acid 3-(2-naphthyl)-l-alanine (NA) underwater adhesive as the multifunctional cathode to meet printable and bendable demands, as well as stability in a high humidity environment (Fig. 11b). They found that the assembled MERAB with HPAs/NA cathode and Al anode changed from a reddish-brown to deep-blue color without any external power. Such a spontaneous phenomenon can be contributed to the reaction of aromatic and water-immiscible NA in a hydrophobic microenvironment. More interestingly, the color can be recovered upon adding H_2_O_2_, as shown in Fig. 11c. It is clear that robust mechanical and environmental durance, together with the strong oxidized capability of H_2_O_2_, offers a promising user-device interactive platform. In general, an external inducer with a strong oxidation ability is an essential factor in realizing self-powered operation. However, the repeated introduction of oxidizing agents can affect the electrolyte concentration, making it difficult to maintain their original performances.

Instead, the utilization of the redox potential difference between the cathode and anode offers another strategy. For example, Zn anode exhibits a much lower charged/bleached potential in comparison with those of Li- and Al-based electrochromic batteries, suggesting a lower energy consumption during the charging process. Due to the multi-valance state of V_3_O_7_, the electrochromic battery assembled by V_3_O_7_ cathode, Zn anode and ZnSO_4_ electrolyte can function as a multicolor display as shown in Fig. 11d (fully yellow, fully grayish blue, and half yellow-half grayish blue) [125]. Such a Zn-V_3_O_7_ MERAB can retrieve the consumed energy for bleaching and realize zero energy consumption for the coloration process. Specifically, an energy density of 15.2 mWh g^−1^ can be retrieved and used to power an LED for 28 min (Fig. 11e). Furthermore, given the great redox potential difference (2.8 V) between Mg/PB, Dong et al. [126] successfully fabricated a fast self-charging and rechargeable Mg/PB MERAB with an open circuit potential of 2.5 V as shown in Fig. 11g, which was higher than that of Al/PB (1.26 V) [23] and Al/WO_3_ (1.96 V) [124]. With a synergic effect of the oxidation capability of NaClO_4_, the self-charging/coloring time of PB is only 6.24 min, which is 480-fold faster than that of the system without NaClO_4_ (Fig. 11h). Based on the above discussion, it can be concluded that the key factor in determining the output performance in different self-powered systems lies in the match between the cathode and anode.

In general, self-powered MERABs as an energy-efficient platform have been applied in energy storage, smart windows, displays, and biosensors [127, 128]. However, the generally poor cycling life and long self-charge time hinder practical applications. The fading in the optical modulation and power density, as well as the charging time, can hardly reach their initial state even after repeated switching. Searching for suitable cathodic or anodic catalysts with rational oxidizers should be given primary importance among priorities to synergistically facilitate electron transfer and redox kinetics.

### From Single Entity to Integrated MERABs System

**Fig. 12:**
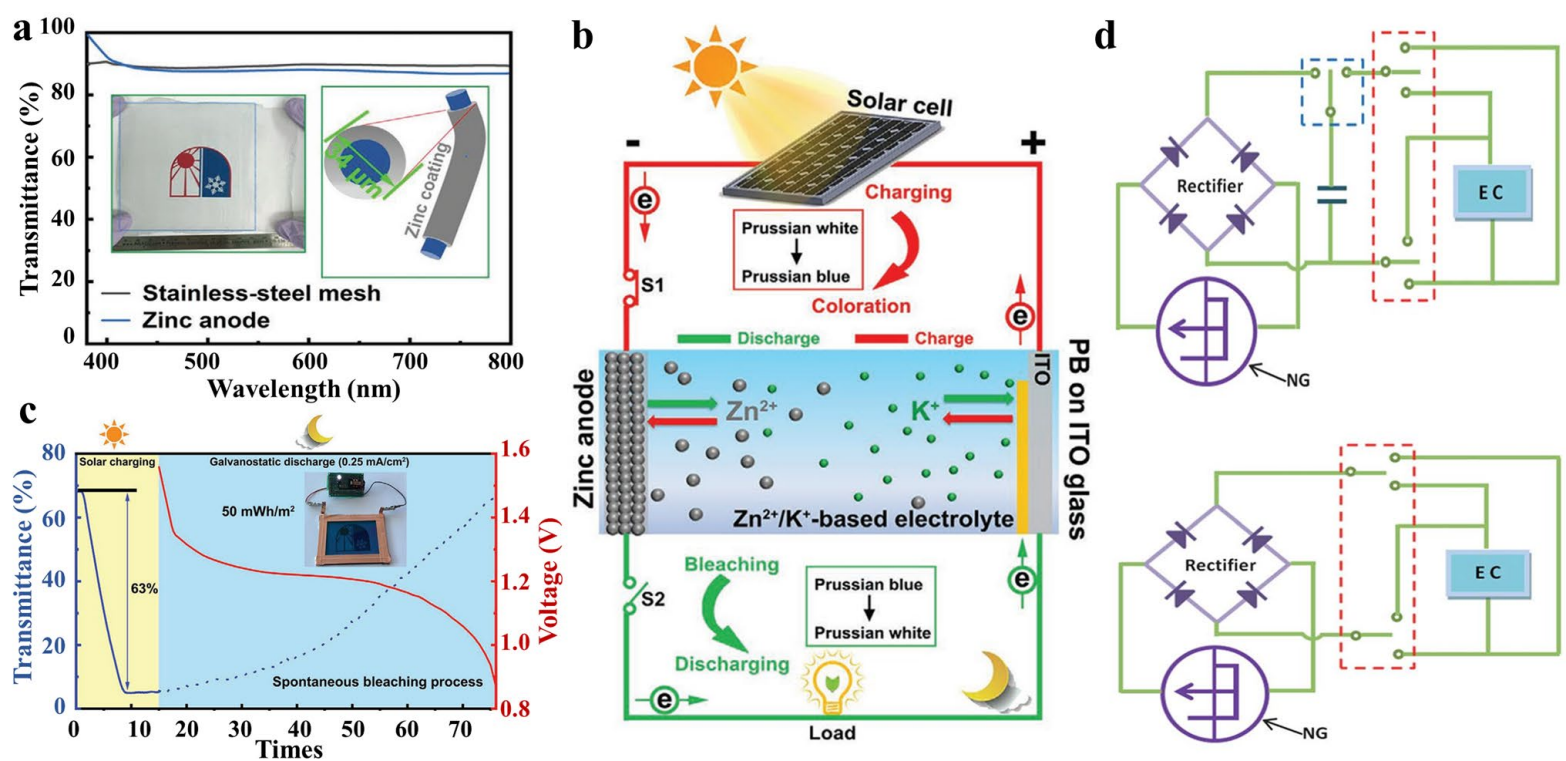
Advances in integrated MERABs system. **a** Optical spectra of the core–shell-structured transparent Zn-mesh. **b** Schematic of the configuration of PV-MERAB and its work mechanism. **c** Optical and galvanostatic curves of the PV-MERAB [35]. Copyright 2020, Wiley–VCH. **d** Nanogenerator-charged power source and real-time power supply. Reproduced with permission [133]. Copyright 2012, Royal Society of Chemistry

Integration of an appropriately designed new function can wholly improve the feasibility of MERABs with multi-functionalities in various fields. Integrating harvesting systems or sensors is an ongoing topic to expand MERAB applications into solar cells, nanogenerators, and healthcare. MERABs integrated with a solar cell not only make use of the abundant solar energy but also solve the self-powered issue for MERABs [129]. However, given the requirement for optical transparency, stable energy output and adequate working voltage, one can see the difficulty in directly combining solar cells with MERABs into one entity [27]. Most of the reported works have focused on optimizing energy conversion/storage systems instead of exploring how to integrate them with MERABs. Energy conversion and storage devices are normally built independent of each other. Therefore, the energy conversion efficiency and output voltage largely determine the electrochemical performance, including the switching time, optical modulation and energy density [130]. Recently, Li et al. [35] proposed an energy-efficient solar-charging MERAB as a smart window in an attempt to address the solar intermittency and self-powered issue. As shown in Fig. 12a, Zn-mesh was used to overcome the limitation of anode opacity and provide a uniform electric field spatial distribution. As presented in Fig. 12b, during the daytime, photovoltaic (PV) cells can supply the necessary electrical power to tint and charge the Zn-MERAB, simultaneously blocking the heat brought from visible and near-infrared light irradiation to cool the indoor temperature. At night, the colored Zn-MERAB can then be spontaneously bleached to power an electrical load due to the redox potential difference. Excellent electrochromic and electrochemical performances (Δ*T* ≈ 63%, *t* = 10 s and 50 mWh m^−2^) have been reported for an intelligent system toward practical applications (Fig. 12c). In addition, mechanical energy as an intermittent and irregular resource can also be harvested into electricity by TENGs [131, 132]. The thus-generated energy can be stored by MERABs to address the issues of associated with the unstable power output of TENGs. Wind-blowing and rain-dropping can also be utilized as resources for wearable electronics, therefore, achieving a high output energy and fast switching speed as demonstrated in Fig. 12d [133]. Other available energy sources in nature, including ocean waves and sounds, are also potential candidates for further research. However, the main challenge in integrating harvesting systems is never based on the question of how to connect such systems together but rather how to incorporate them into a single unit with multi-functionality. This review summarizes the main progress regarding the conversion efficiency of integrated MERABs systems and their partiners, integrated supercapacitor/battery systems (Table 1). Conversion efficiency in a multisystem can be rather low, and thus optimizing the conversion efficiency is a priority consideration, in comparison with the single entity.

**Table 1:** A summary of the conversion efficiency of integrated MERABs systems and their similar integrated supercapacitor/battery systems

Type	Energy storage materials	Energy conversion	Energy efficiency (%)
MERABs	Prussian blue [131]	Sound to electricity	59.85
	V_2_O_5_/P3HT/rGO [134]	Solar to electricity	1.2
	WO_3_-(TiO_2_)-CdS [135]	Solar to electricity	0.3
	PEDOT:PSS/P3HT [136]	Solar to electricity	1.57
Solar cells-supercapacitor	Ti/TiO_2_/CNTs [137]	Solar to electricity	2.73
Solar cells-supercapacitor	TiO_2_ [138]	Solar to electricity	1.64
Solar cells- Li-ion battery	TiO_2_/LiCoO_2_ [139]	Solar to electricity	0.82

**Fig. 13:**
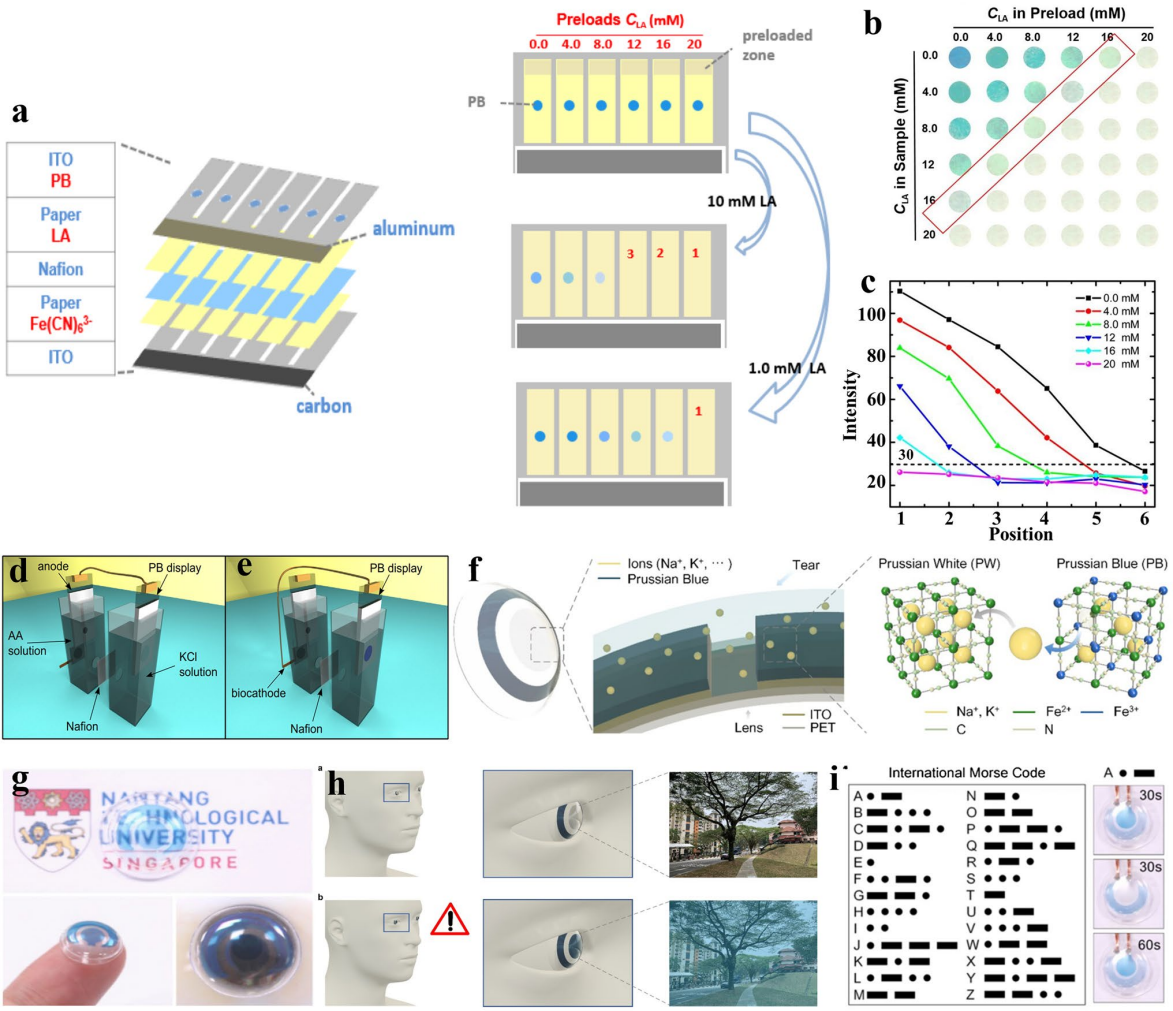
Advances in MERABs sensors. **a** Schematic illustration of the structure and working principle. **b** Photographs of the PB spots on the LA electrochromic array. **c** Color intensity of the PB spots shown in **b** as a function of LA concentration. Reproduced with permission [33]. Copyright 2017, MDPI. **d, e** Schematic illustration of a self-powered biosensor. Reproduced with permission [140]. Copyright 2014, Elsevier. **f** Schematic image of electrochromic alarm system on contact lens. **g** Digital photographs of electrochromic contact lens. **h** Transparent vision on smart contact lens in the safe situation, and color changing in the emergency situation. **i** Morse code in electrochromic alarming system. By controlling the time duration of color change. Reproduced with permission [34]. Copyright 2020, Elsevier

In addition to integration with energy harvesting systems to aim for the self-powered feature, integrating sensors with MERABs is of great interest in portable and wearable devices due to the unique color-changing characteristics [140–142]. To date, the demand for point-of-care testing (POCT) has promoted the development of sensors with semi-quantitatively analysis. Liu et al. [33] reported an integrated paper-based electrochemical sensing system that consists of six parallel electrochromic cells powered by an aluminum-air battery as shown in Fig. 13a. In detail, paper-based detection reservoirs with different amounts of lactic acid (LA) act as the analyte and electrolyte, and PB was employed as an electrochromic indicator. Notably, PB can be reduced to colorless PW, when the concentration of LA reached a set value, and a higher concentration can induce the generation of more color-changing spots (Fig. 13b, c). Niedziolka et al. [140] have further complemented self-powered function into sensors. An ascorbic acid/O_2_ biofuel cell and PB electrochromic display was fabricated and used to confirm the concentration of ascorbic acid in juice drinks (Fig. 13d, e). Therefore, the conversion from electrochemical signals to visual readouts was successfully built. In the future, for a suitable choice of oxidase, the basic prototype can be extended to detect other types of analytes, such as glucose and uric acid. Another interesting application is for real-time smart healthcare systems. A smart contact lens based on MERABs enables vital-sign monitoring through enhanced visual information that would be utilized for early warning and telecommunication. Lee et al. [34] employed PB films on the cornea above the pupil, together with tears as an aqueous electrolyte, to alter the brightness of vision for users (Fig. 13f). The connection between the simulated signal and brightness controlled by an external voltage is built on MREABs and biosensors. Generally, these smart contact lenses are transparent, but they receive biosensor signals and simultaneously show blue to call for attention when in an emergency state (Fig. 13g, h). More interestingly, the Morse code for international telecommunication encoding typically involves the transmission two distinct signal durations, which can be applied in smart contact lenses by adjusting the duration time of the applied voltage (Fig. 13i). In this way, confidential information, as well as guidance information for people with hearing difficulty, can be visually presented by the contact lens. The configuration and materials of these electrochromic contact lenses are almost the same as those used in aqueous batteries; therefore, it would be likely and valuable to combine an aqueous battery with the smart contact lens to realize external power-free devices in the future. It is believed that the MREAB-biosensor platform will provide a novel avenue in real-time monitoring, information interaction, and data visualization.

In general, an integrated system endows MERABs with more functionalities but leads to more demands for design and fabrication. There is a general requirement for a simplified structure and lightweight in practical applications. An integration of multi-functionalities shall thus consider the compatibility of different working principles; therefore, searching for adequate materials systems and optimizing the device configuration is the apparent indicator in future research.

### From Independent System to Multi-system Conversion

**Fig. 14:**
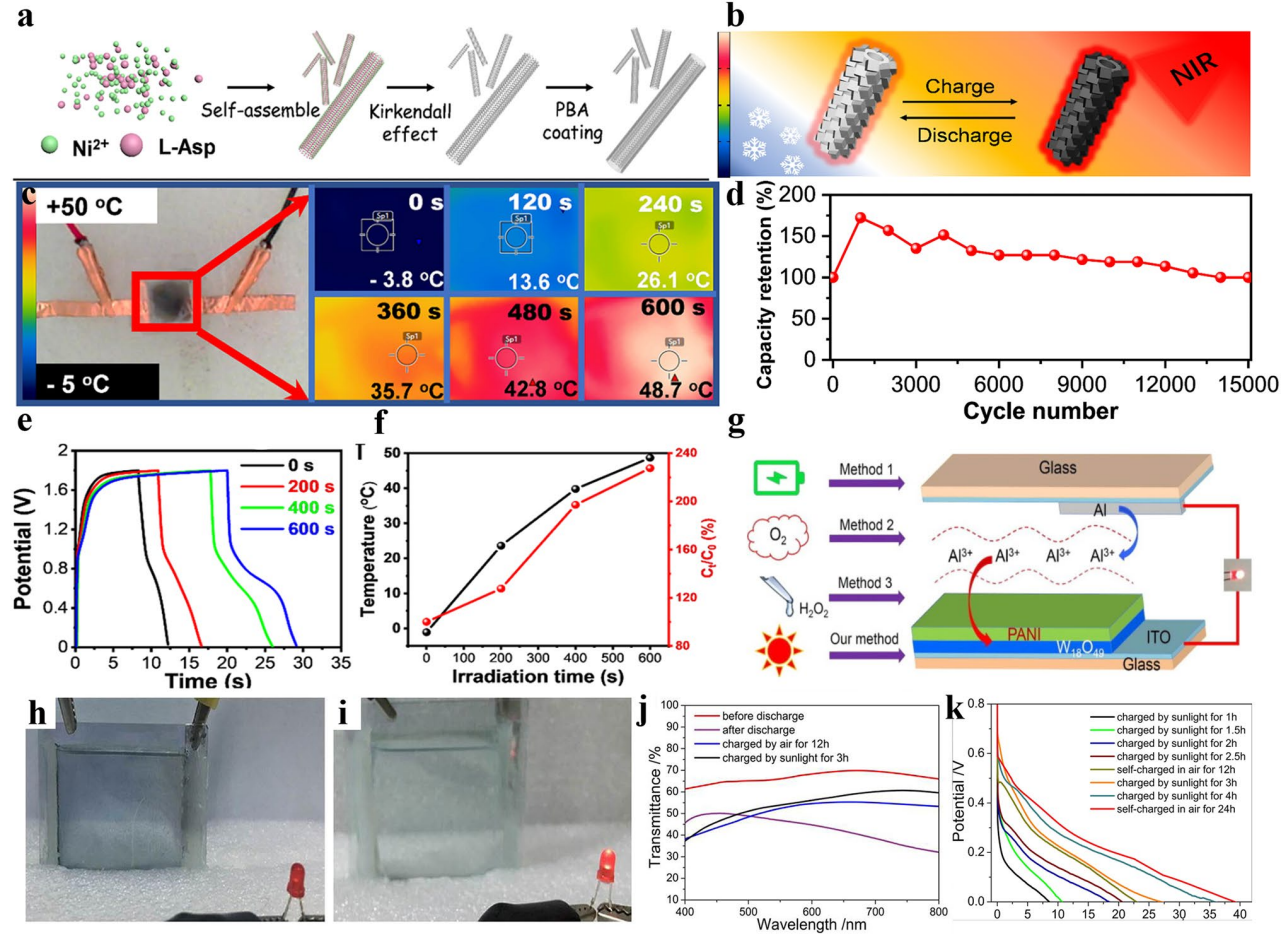
Advances in multi-system conversion for MERABs. **a** Schematic of the preparation process of PBA/NiO electrode. **b** Schematic of electrochromical thermal-tuning effect of PBA/NiO electrode. **c** Infrared images of the PBA/NiO-based device under different solar irradiation times. **d** Cycling performance. **e** GCD curves. **f** Temperature and specific capacitance percentage under different solar irradiation times. Reproduced with permission [36]. Copyright 2021, American Chemical Society. **g** Schematic of charging routes for W_18_O_49_/PANI electrochromic battery. **h** W_18_O_49_/PANI electrochromic battery in colored state with energy exhausted. **i** W_18_O_49_/PANI electrochromic battery charged by sunlight irradiation for 3 h. **j** In-situ transmittance spectra, and **k** GCD curves by different charge methods. Reproduced with permission [152]. Copyright 2018, Elsevier

In current MERABs, the high freezing point of water presents a critical drawback for aqueous batteries, especially in a low-temperature environment [143–145]. Introducing appropriate anti-freezing additives into electrolytes has been normally used to suppress the freezing of water [146–148]. Organic solvents (e.g., ethylene glycol, dimethyl sulfoxide, and glycerol) are common additives, which can interact with water molecules to suppress water freezing. However, the decreased ionic conductivity and flammable risks may not be avoided. As a comparison, employing “water-in-salt electrolyte’’ (WiSE) is also a promising strategy, where the inorganic salts exceed the solvent in both weight and volume. Although these strategies greatly decrease the freezing point of electrolytes, the enlarged voltage window might not be suitable for some electrodes. Interestingly, a new route is examined for improving device endurance in a harsh environment that is modulating photo-thermal conversion in MERABs to fight against low temperature. In this regard, the device temperature can be quickly recovered after several minutes of exposure to sunshine due to its high absorption ability in the solar radiation spectrum. As shown in Fig. 14a, Lu et al. [36] deposited PBA onto NiO nanotubes as the functional electrode. Benefiting from the excellent light-to-heat conversion properties and fast intercalation/deintercalation capacity of PBA, the local surface temperature rapidly increases from -4.0 to 45.6 °C in a short irradiation period of 30 min (Fig. 14b, c). As a result, it can deliver a capacitance increase by 377.8%, as well as an ultra-long cycling performance of 15,000 cycles (Fig. 14d). According to the GCD curves (Fig. 14e), the specific capacitance of the prototype increases from 24.3 to 55.2 F g^−1^ after being exposed to solar irradiation for 10 min as shown in Fig. 14f. Similar work has also been reported for supercapacitors with Cu_1.5_Mn_1.5_O_4_ [149] and graphene [150]. Based on previous experience gained from the study of thermally manipulated supercapacitors, metal oxides, such as TiO_2_, WO_3_, and NbO_*x*_, with a tunable localized surface plasmon resonance (LSPR) can also be used to obtain satisfactory photo-thermal conversion [151]. Furthermore, the high thermal conductivity of the metal anode can effectively lead to a temperature increase. Therefore, solar thermal energy could act as a renewable technology that inspires new insights for MERABs in cold environments.

In addition, photo-thermal conversion can also meet self-powered requirements. An external inducer with strong oxidation ability is an essential factor to realize self-powered operation, where O_2_ and H_2_O_2_ are representative examples. Chemical charging of MERABs holds the merits of efficient reaction rate, but the unavoidable side reaction between the metal anode and additives can lead to performance fading for MERABs [152]. In comparison, light charging is environmentally friendly and convenient for powering the exhausted MERABs [134, 135, 153]. Chang et al. [152] developed a sunlight-charged MERAB based on a W_18_O_49_@PANI cathode and Al anode (Fig. 14g). The charging rate could be significantly enhanced by light irradiation as shown in Fig. 14h, i. As a result, the charging time for a W_18_O_49_@PANI/Al MERAB can be effectively reduced by six times compared with that for the chemical-charging process. In addition, the discharge capacity can reach 36.78 mAh g^−1^ after 4 h of irradiation. The same level can only be reached after being exposed to air for 24 h. Additionally, the transmittance at 700 nm was increased to 60.1% after being exposed to sunlight irradiation for 3 h, which is much higher than that (55%) obtained for self-chemical charging in air for 12 h (Fig. 14j, k). In detail, light irradiation promoted the oxidation reaction of the W_18_O_49_@PANI and facilitated ions diffusion in the AlCl_3_ aqueous electrolyte. Furthermore, there is the occurrence of chemical bonding betweenW_18_O_49_ and PANI that benefits the charge transfer. Photo-excited electrons are generated in the conduction band of W_18_O_49_ with light irradiation, which reduces PNAI from ES (conductive) to LS (insulating). In the future development, efficiently compositing of high-conductivity materials to accelerate charging and switching speed can be an interesting pursuit.

**Fig. 15:**
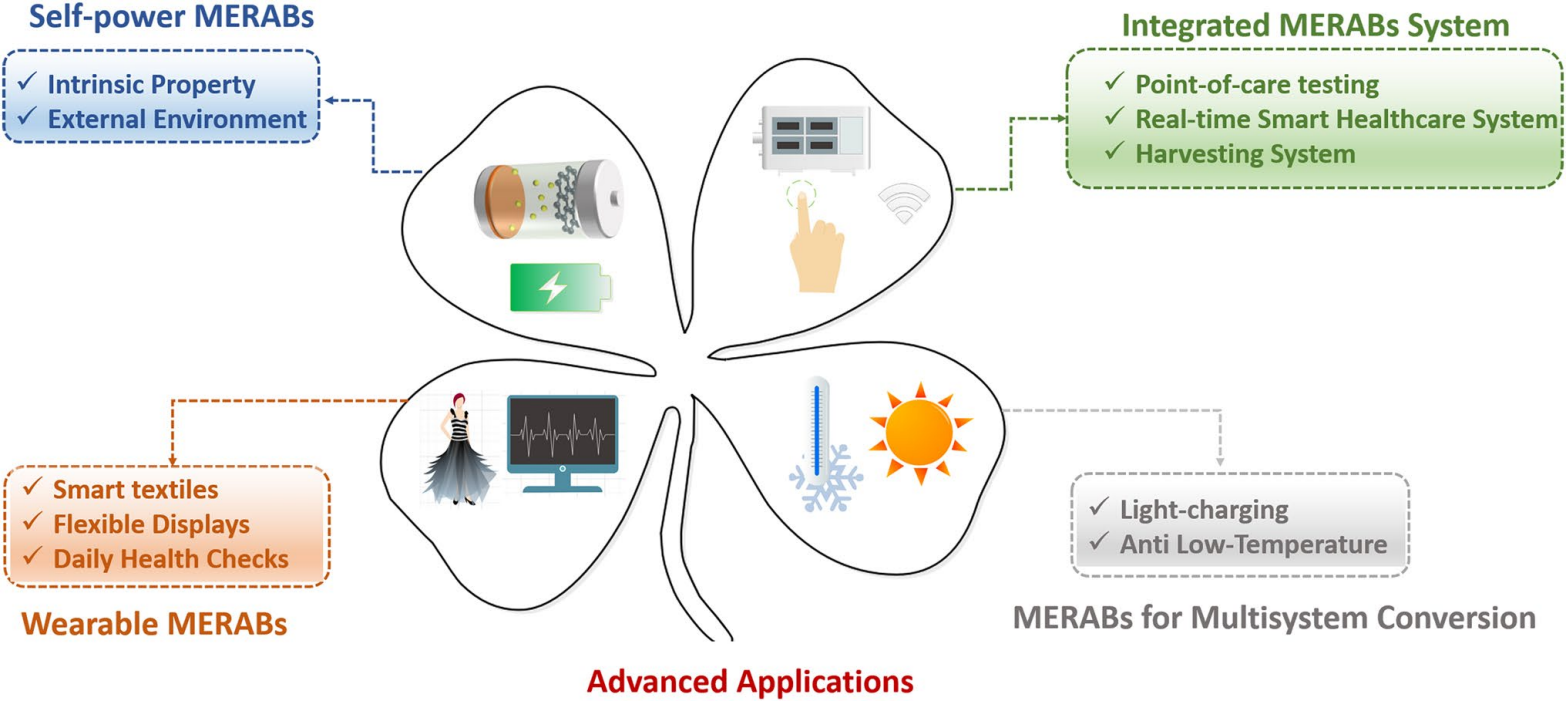
Summary of recent advances in MERABs

Overall, different novel MERABs have been emerging in the last decade. There are many other functions and applied scenarios that need to be further explored. The four main types of MERABs with their potential applications are summarized in Fig. 15.

## Conclusions and Future Challenges

**Fig. 16:**
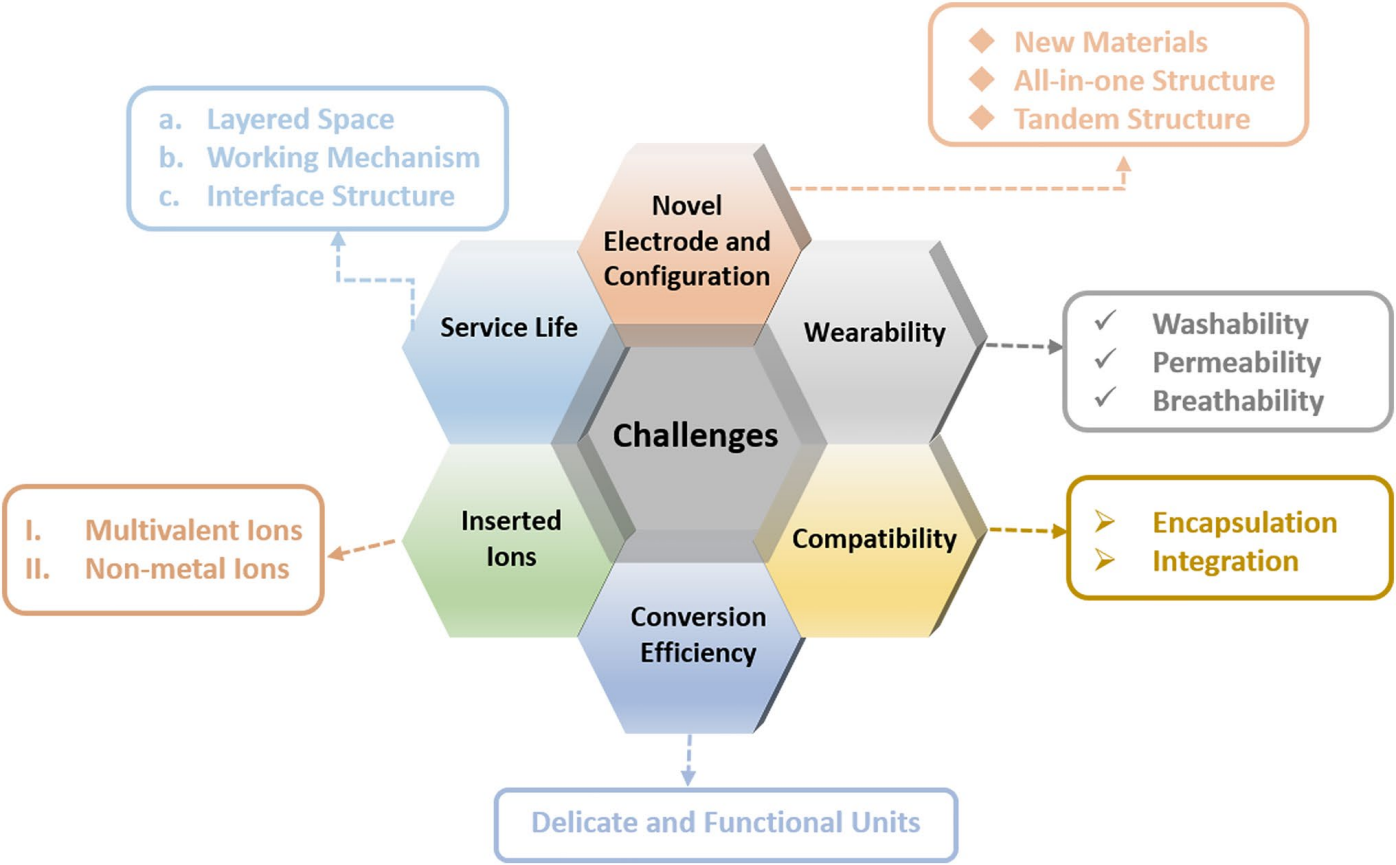
Schematic diagram of main challenges and development trend of MERABs

In summary, MERABs are able to combine the strengths of aqueous ion batteries and electrochromism to realize conversion and storage in photo-thermal-electrochemical multi-systems. Electrochromic devices are capable of dynamically modulating solar light and heat radiation but with generally sluggish reaction kinetics and low storage capacity. The unique advantages of power density and high ionic conductivity of aqueous ion batteries can well compensate for these shortcomings. Apart from the basic energy storage characteristics, the additional functionalities of wearability, visualization, self-powered operation, low-temperature resistance, etc. can be incorporated into MREABs. In this review, the underlying mechanisms ranging from materials principles, device designs to advanced applications are comprehensively discussed. The main challenges and development trends are summarized in Fig. 16. Although great progress has been achieved, the development of MERABs is still far from ready for large-scale practical applications. Several challenges in the instability, unsatisfactory storage capacity, and low conversion efficiency need to be addressed.

1. *Searching for novel MERABs electrode materials*. The most common MERAB electrode materials are tungsten oxide, titanium dioxide, PANI and PB. MERABs based on these materials show power densities of the MERABs that are generally lower than 4 W m^−2^ [105, 125, 154, 155], which cannot match that obtained using conventional ion batteries. Ideally, electrochromic materials should require a smaller charge density to induce a larger optical density, thereby obtaining an excellent responsive ability under a narrow potential window. However, battery materials are expected to pursue high energy storage capacity under a wide potential window. Therefore, the development of a novel MERAB material is essential to improve the energy/power density and balance the trade-off between electrochromic and battery performance.
2. *Optimizing the device configuration*. Typical MERABs consist of five-layered structures including two conducting layers, an anode, an electrolyte and a cathode. This rather complicated multilayered configuration can unavoidably lead to a high interfacial impedance and an ambiguous transportation path for electrons/ions. To address this issue, the following approaches can be considered: (i) An “all-in-one” architecture, where more specifically, the combination of electrode materials and redox mediators into electrolyte layers can reduce the diffusion length for ions and eliminate the charge barrier resulting from the interface. The redox reaction that occurs between the electrode materials and redox mediator guarantees charge balance. Notably, it will be necessary to employ soluble electrode materials. (ii) A tandem structure. For example, Cao et al. [156] deposited a solid electrolyte layer on top of PEDOT:PSS and WO_3_ electrode and also below a PEDOT:PSS electrode. The electrolyte provides metal ions to PEDOT:PSS and pumps protons to WO_3_. Moreover, the insulating electrolyte layer endures a high voltage to avoid the generation of H_2_ in the PEDOT:PSS electrode. The tandem structure also enables different ions to diffuse effectively and sequentially.
3. *Improving the compatibility of functional components*. The performance of a single component can hardly determine the performance of the whole device. The encapsulation process and the matching of the cathode, anode and electrolyte are also key factors to obtain high performance. For example, WO_3_ or PBA are more suitable than NiO or TiO_2_ in aqueous Zn^2+^ electrolytes. More specifically, the lattice expansion of the host materials and diffusion energy barrier for ions should be considered.
4. *Optimizing the conversion efficiency between different thermal-photo-electrochemical systems*. Integration of harvesting systems or sensors is an ongoing topic to expand MERAB applications. A direct connection of the functional units and MERABs is a facile strategy but is often limited by the conversion efficiency of different systems, such as the solar-electricity and mechanical-electricity efficiencies. Furthermore, the output voltage should meet the driving voltage of the MERABs. Another approach is to design a multifunctional unit for integration into one entity. The advantages of high efficiency without any intermediate processes and lightweight shall be more suitable for next-generation flexible electronics. In this regard, investigation into the delicate fabrication and working mechanisms is challenging to realize integrated MERABs systems in the future.
5. *Designing suitable ion carriers*. Multivalent intercalation ions owing to their multi-electron carriers, high capacity and natural abundance are widely applied in various functional devices. For example, Zn^2+^ ions show low redox potential (− 0.76 V vs. SHE), and Mg^2+^ ions possess a high theoretical volumetric energy density of 3866 mAh cm^−3^, and Al^3+^ ions support the three-electron redox reactions [157–159]. However, avoiding the lattice expansion of host materials and corrosion of metal anodes resulting from strong electrostatic interactions and polarization are among the prior considerations. Additionally, a wide potential window is the basic requirement for high energy/power density. On the other hand, nonmetal ions can also be appealing. For example, the ammonium (NH_4_^+^) ion, as a representative example, possesses a special peculiar tetrahedral structure without a preferred orientation. Moreover, the advantages of less corrosion and high dissociation of NH_4_^+^ make it a promising aqueous electrolyte ion for MERABs. The thickness of the electrolyte layer and electrode layer should be reduced, which will benefit the transmittance and enable adaptation to the portable and flexible era.
6. *Improving comfortability and wearability*. Wearability is a vital characteristic in shaping and improving human life quality. Wearable MERABs can not only be weaved into fabrics but also be applied in VR smart glass or contact lens. Thus, non-toxicity, safety, comfortability and tailorability shall be realized. Noted that repeated washability, permeability and breathability shall be considered in fiber-based wearable MERABs.
7. *Prolonging the service life of devices*. High stability is the basic requirement in any practical application. However, most of the known MERABs can only endure several thousand cycles. To address this issue, three approaches can be considered. (i) Understanding the working mechanism, especially on those of multivalent ions. (ii) Constructing a robust interface structure. We have previously shown that the stabilization of the host material lattice by *in-situ* grown composites to obtain a robust interface structure. The formation of chemical bonds at the interface ensures lattice integrity, thereby improving the cycling life [21, 43, 83]. (iii) Expansion of the layered space offers another approach. Introducing organic additives during the synthesis process, such as PVP and PEO, to interact bonds with monolayers [96, 160]. The enhanced diffusion kinetics and stable interlayer structure shall be materialized.
8. *Advanced manufacturing*. Advanced manufacturing, including packaging and film-forming technology, are basic for the electrochromic/electrochemical performances. Specifically, as an alternative of traditional sputtering deposition process, all-solution deposition methods (e.g., roll-to-roll method, spray coating, 3D printing and so on) have been demonstrated in industry with low-cost and high-quality characteristics, which will help promote the translation of MERABs from laboratory to industry [49]. In addition, suitable packaging materials provide oxygen- and water-proof, ensuring the high stability of MERABs. Particularly, emerging liquid metals (LMs) possess both metallic and fluidic properties, which shall provide an opportunity for MERABs to achieve the desired stretchable and hermetic sealing [161].

Although conventional aqueous batteries offer an excellent energy storage capability, single-functionality batteries cannot well satisfy the requirements for various application scenarios. In contrast, new MERABs incorporating electrochromism into aqueous batteries shall be able to realize the integration of photo-thermal-electrochemical multidisciplinary. MERABs bring promising potential and great challenges at the same time for emerging functional energy devices. It is envisioned that with the numerous new efforts being devoted, a balance of excellent electrochromic/electrochemical performance, low cost and large scalability could be realized and that a shift from the laboratory to market would be coming soon.
